# Modeling Ligand Exchange Kinetics in Iridium Complexes Catalyzing SABRE Nuclear Spin Hyperpolarization

**DOI:** 10.1021/acs.analchem.4c01374

**Published:** 2024-07-08

**Authors:** Oleg G. Salnikov, Charbel D. Assaf, Anna P. Yi, Simon B. Duckett, Eduard Y. Chekmenev, Jan-Bernd Hövener, Igor V. Koptyug, Andrey N. Pravdivtsev

**Affiliations:** † International Tomography Center SB RAS, 3A Institutskaya St., 630090 Novosibirsk, Russia; ‡ Section Biomedical Imaging, Molecular Imaging North Competence Center (MOIN CC), Department of Radiology and Neuroradiology, University Medical Center Kiel, Kiel University, Am Botanischen Garten 14, 24118 Kiel, Germany; § Novosibirsk State University, 2 Pirogova St., 630090 Novosibirsk, Russia; ∥ Centre for Hyperpolarization in Magnetic Resonance (CHyM), University of York, Heslington YO10 5NY, U.K.; ⊥ Department of Chemistry, Integrative Biosciences (Ibio), Karmanos Cancer Institute (KCI), Wayne State University, Detroit, Michigan 48202, United States

## Abstract

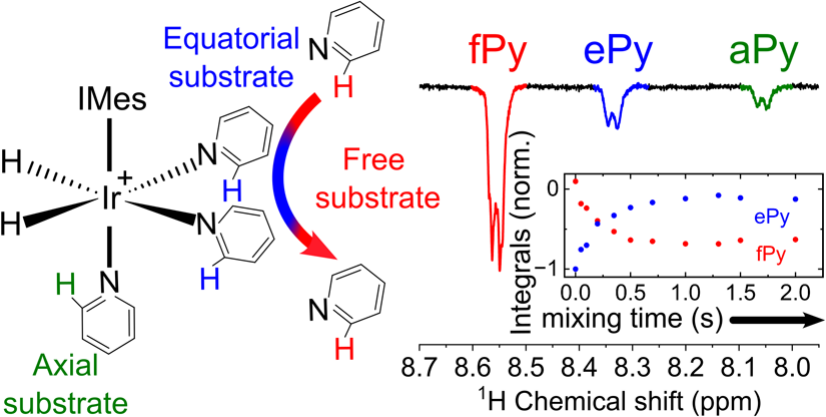

Large signal enhancements can be obtained for NMR analytes using the process of nuclear spin hyperpolarization. Organometallic complexes that bind parahydrogen can themselves become hyperpolarized. Moreover, if parahydrogen and a to-be-hyperpolarized analyte undergo chemical exchange with the organometallic complex it is possible to catalytically sensitize the detection of the analyte via hyperpolarization transfer through spin–spin coupling in this organometallic complex. This process is called Signal Amplification By Reversible Exchange (SABRE). Signal intensity gains of several orders of magnitude can thus be created for various compounds in seconds. The chemical exchange processes play a defining role in controlling the efficiency of SABRE because the lifetime of the complex must match the spin–spin couplings. Here, we show how analyte dissociation rates in the key model substrates pyridine (the simplest six-membered heterocycle), 4-aminopyridine (a drug), and nicotinamide (an essential vitamin biomolecule) can be examined. This is achieved for the most widely employed SABRE motif that is based on IrIMes-derived catalysts by ^1^H 1D and 2D exchange NMR spectroscopy techniques. Several kinetic models are evaluated for their accuracy and simplicity. By incorporating variable temperature analysis, the data yields key enthalpies and entropies of activation that are critical for understanding the underlying SABRE catalyst properties and subsequently optimizing behavior through rational chemical design. While several studies of chemical exchange in SABRE have been reported, this work also aims to establish a toolkit on how to quantify chemical exchange in SABRE and ensure that data can be compared reliably.

## Introduction

**Figure 1:**
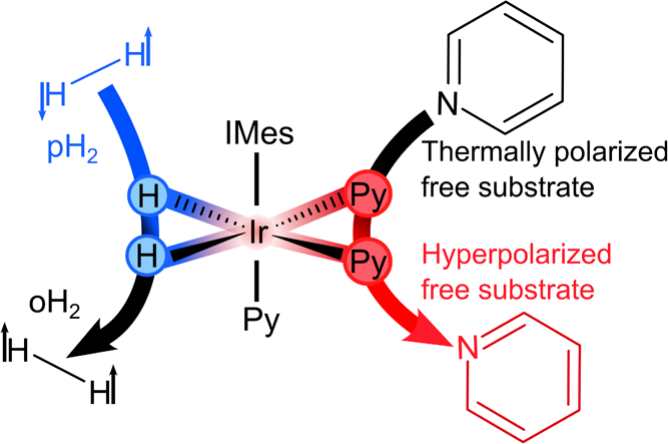
Scheme of SABRE hyperpolarization of pyridine. pH_2_ and a substrate (here: pyridine, Py) bind to form a transient complex, [Ir(H)_2_(IMes)(Py)_3_]Cl. Spin–spin interactions then drive spin order from IrHH (the pH_2_-derived hydride spins) to the Py substrate, resulting in two hyperpolarized equatorial substrate ligands (red Py) and orthohydrogen (oH_2_). IMes stands for 1,3-bis(2,4,6-trimethylphenyl)imidazole-2-ylidene. The two equatorial chemically equivalent pyridine ligands exchange via a dissociative (S_N_1) mechanism.^22^

Nuclear spin hyperpolarization techniques are reshaping the field of NMR spectroscopy and imaging by dramatically enhancing sensitivity.^1^ Dissolution dynamic nuclear polarization (dDNP)^2^ employs high thermal equilibrium polarization of electron spins at low temperatures and high magnetic fields as a source of hyperpolarization, with polarization transfer induced by the application of microwave irradiation of the solid sample with a subsequent rapid sample dissolution.^3^ As a result, the dDNP technique requires expensive and complex equipment. Alternatively, the sensetivity of liquid-state NMR spectroscopy can be enhanced using the singlet nuclear spin isomer of dihydrogen (parahydrogen, pH_2_) as a hyperpolarization source.^4^ Here, hyperpolarization is often achieved by pairwise addition of pH_2_ to an unsaturated substrate in the parahydrogen-induced polarization (PHIP)^5,6^ experiment, or by transient coordination of both pH_2_ and a substrate to an organometallic complex in the signal amplification by reversible exchange (SABRE) experiment (Figure 1).^7,8^ In the latter case, the transiently formed organometallic complex enables polarization transfer from the nascent H atoms that originate from pH_2_ to the nuclear spins of the coordinated substrate. This polarization transfer can be achieved spontaneously (by free evolution in an appropriate magnetic field)^7^ or driven by specially designed radiofrequency (RF) pulse sequences.^9^

Compared to conventional hydrogenative PHIP, an advantage of SABRE is that the substrate is chemically not modified during the process. As a result, this approach has substantially broadened the range of compounds that can be polarized using pH_2_.^10−14^ Moreover, the analyte can be rehyperpolarized multiple times or even continuously, allowing one to obtain useful information about relaxation dynamics, optimize the hyperpolarization process, and perform multidimensional NMR studies^15−17^ under high atom economy. The SABRE hyperpolarization technique has emerged as a valuable tool for producing HP contrast agents and improving NMR spectroscopic characterizations for various analytes and complex biological mixtures.^18−20^

Since the inception of the SABRE effect, great efforts have been put into understanding the interplay between a chemical exchange, a lifetime of the transient complexes involved in catalysis, and the underlying and often weak nuclear spin–spin interactions.^21−27^ Unfortunately, these nuclear spin–spin interactions have proven challenging to quantify (because of the chemical exchange). Adjusting the couplings in a controlled manner is not possible either. The lifetime of the complex and the related chemical exchange processes are easily modified, for example, by temperature variation^28−30^ or by varying the electronic and steric properties of the ligands surrounding the metal center.^31^

Measuring the exchange kinetics is possible by classical NMR methods like ^1^H or X-nuclear exchange spectroscopy (EXSY),^32,34,35^ as introduced by Ernst et al.,^32^ or its 1D variant with selective excitation (SEXSY).^36,37^ Alternatively, the lifetime of the complexes can be measured indirectly by observing the effect of chemical exchange on polarization transfer efficiency.^28,33^ Although previous SABRE studies often report substrate exchange rates, the experimental and theoretical approaches used to rationalize them differ.^10,21,28,32^ In practice, the most rapid measurements with higher sensitivity and more evolution time steps can be achieved through the SEXSY variant. Alternatively, heteronuclear SABRE exchange kinetics can be measured with superior signal strengths by delay variation in INEPT-based RF pulse sequences used for polarization transfer from hydrides to the substrate nuclei.^24,35^

Herein, we compared three substrate exchange kinetic models of different complexity that map onto experimental observations obtained with EXSY and SEXSY. This study involved the examination of the dynamic behavior of [Ir(H)_2_(IMes)(substrate)_3_]Cl complexes and the SABRE exchange rates for three substrates: pyridine (Py), 4-aminopyridine (4AP), and nicotinamide (NAM). Hence, by utilization of the most widely employed SABRE precatalyst [IrCl(COD)(IMes)],^10^ these data will be relevant to many workers in the area that is already translating into *in vivo* applications and the analysis of complex mixtures.^38,39^

Our rationale for choosing these substrates was to reflect the important role related six-membered heterocycle motifs play in a wide range of drugs and biologically relevant molecules, many of which have already been shown to be amenable to SABRE hyperpolarization. Specifically, 4AP is a drug used in the symptomatic treatment of multiple sclerosis,^40^ while NAM is representative of the biomolecule vitamin B3. We anticipate that the results of this study will apply to the much wider range of analytes used in SABRE, including those used in the analysis of complex mixtures^18−20^ and the production of HP contrast media such as [1-^13^C]pyruvate;^41−43^ this material is under evaluation as a probe to image a wide range of diseases in over 50 clinical trials according to clinicaltrials.gov.

## Methods

### Chemicals

The Ir precatalyst ([Ir] = [IrCl(COD)(IMes)]; IMes = 1,3-bis(2,4,6-trimethylphenyl)imidazol-2-ylidene, COD = 1,5-cyclooctadiene) was synthesized according to ref.^10^ Pyridine (Py, Reakhim, > 98%), 4-aminopyridine (4AP, Sigma-Aldrich, 98%), nicotinamide (NAM, Sigma-Aldrich, ≥ 98%), methanol-*d*_*4*_ (Zeotope, 99.8% D) and hydrogen (>99.999%) were used without additional purification.

### Sample Preparation

For all NMR experiments, the samples were prepared by mixing one of the three SABRE substrates (Py, 4AP, or NAM) at a concentration of 40 mM (for Py) or 80 mM (for 4AP and NAM) with 4.0 mM of [Ir] in 600 μL of methanol-*d*_4_.

### NMR Measurements

Each sample was supplied with H_2_ at 15 standard cubic centimeters per minute (sccm) gas flow rate, 7.9 bar, and room temperature until the SABRE precatalyst was converted completely into the SABRE-active dihydride complex, according to ^1^H NMR spectroscopy (25 min for Py, 30 min for NAM, 120 min for 4AP). Next, the sample was depressurized, and the catheter used to supply H_2_ to the solution was pulled up out of the solution by ∼12 cm (still inside the NMR tube), followed by reinitiation of H_2_ flow through the catheter. As a result, the sample resided under an H_2_ atmosphere at ambient pressure during the measurements; at the same time, gradual solvent evaporation was avoided since the gas flowed several centimeters above the solution.

**Figure 2:**
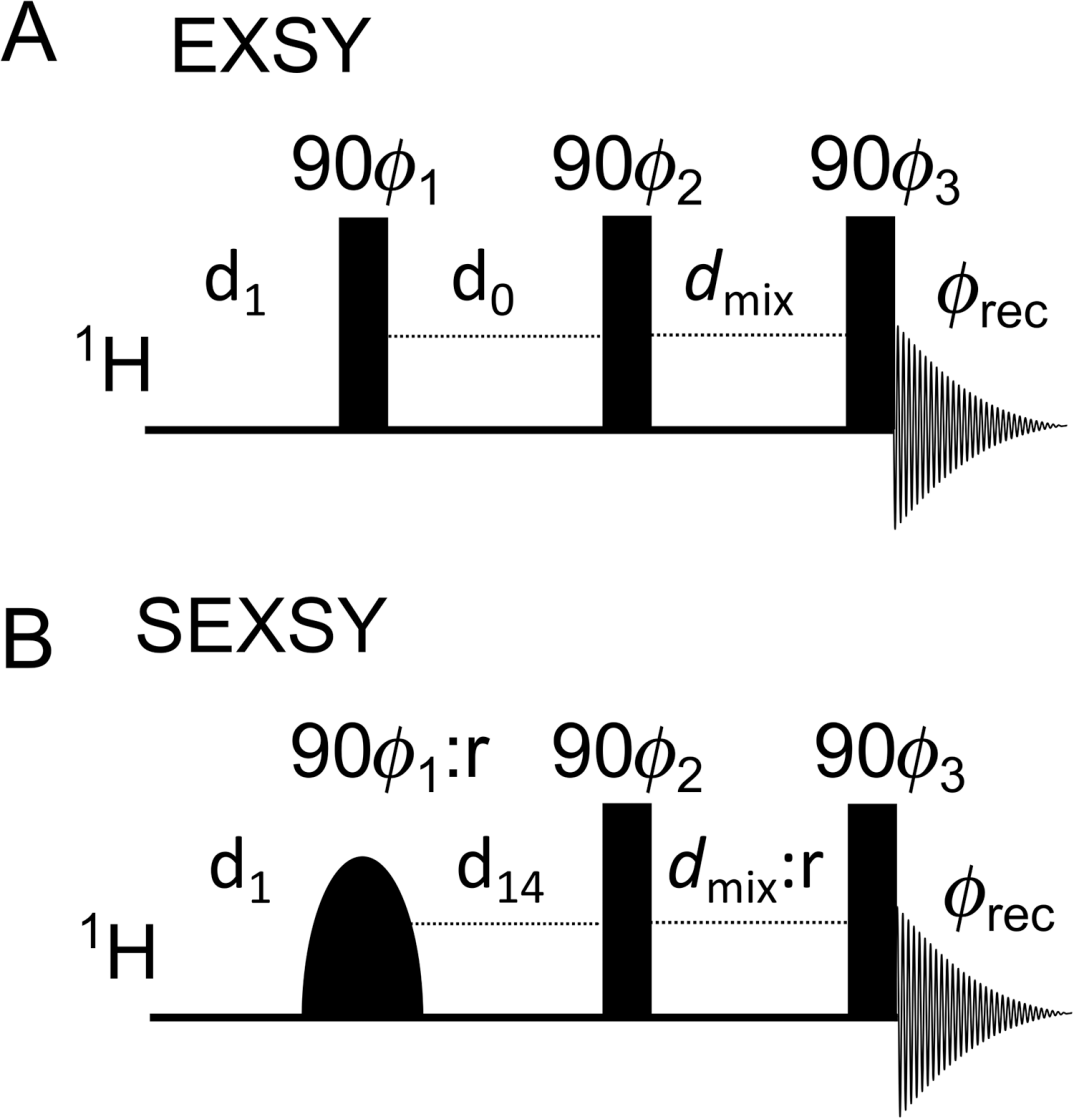
EXSY (A) and SEXSY (B) NMR pulse sequences. The rounded pulse in the diagram is frequency-selective. Several delays were applied: *d*_1_ stands for relaxation delay, *d*_0_ is indirect dimension encoding delay, *d*_14_ stands for evolution after shaped pulse, and *d*_mix_ (typically *d*_8_ in TopSpin) stands for mixing time. Phases: φ_1_ = [0°, 180°], φ_2_ = 0°, and φ_3_ = [0°, 0°, 180°, 180°, 90°, 90°, 270°, 270°], φ_rec_ = [0°, 180°, 180°, 0°, 90°, 270°, 270°, 90°]. In SEXSY sequence, the phase φ_1_ and *d*_mix_ were selected with a random deviation (: r) up to 5%.

NMR spectra were acquired on a 7.05 T Bruker AV 300 NMR spectrometer at several specified temperatures with a 5 mm probe without gradients. At each temperature, the following ^1^H NMR spectra were recorded: a 2D EXSY spectrum with a mixing time *d*_mix_ (using Bruker TopSpin *noesyph* pulse sequence, Figure 2A), a regular 1D spectrum (*zg* Bruker TopSpin sequence), and several 1D selective EXSY (SEXSY) spectra with variable mixing times (*d*_mix_ here is the variable *d8* in a *selno* Bruker TopSpin pulse sequence, Figure 2B). The selective RF excitation (90° Gauss-shaped pulse, 40 ms, corresponding to 52.5 Hz excitation bandwidth) was tuned to excite the frequency of equatorially bound substrate protons (α-protons for Py, H-5 protons for NAM, and β-protons for 4AP). Hence, the corresponding resonances for the equilibrium concentration of the free materials were initially unencoded, and they only became visible through chemical exchange.

For T_1_ relaxation measurements, the sample preparation and handling procedures were the same as described above, except that the iridium catalyst was not added to the solution. The samples were bubbled with H_2_ at 15 sccm, 7.9 bar, at room temperature for 30–50 min to displace any dissolved air. T_1_ was measured using conventional inversion recovery sequence (*t1ir* sequence in Bruker TopSpin).

Corresponding chemical shifts, T_1_ relaxation times of protons of these substrates, and detailed acquisition parameters are provided in Supporting Information (SI).

### Data Processing

All spectra were analyzed using spectral data analyzing software Bruker TopSpin (4.0.7), Bruker Dynamics Center (2.5.5), MestReNova (14.2.2), and Origin (2021). Data were modeled using Origin or the MATLAB (R2021a) MOIN spin-library^24^ as described in the text. The standard deviation for dissociation rates *k*_d_ of SEXSY experimental data obtained using models C_S_S_2_↔C_S_S+S and C_S_S_2_↔S_2_ are calculated using the MATLAB nonlinear regression function “nlinfit”. For EXSY experimental data using model C_S_S_2_↔S_2_, dissociation rate deviation was calculated using eq 32 of ref.^44^ As instructed, the intensity variances have been estimated by assuming a precision (noise and errors due to signal overlapping) of 10% for diagonal and cross peaks.

## Results and Discussion

### Chemical Exchange Models

**Figure 3:**
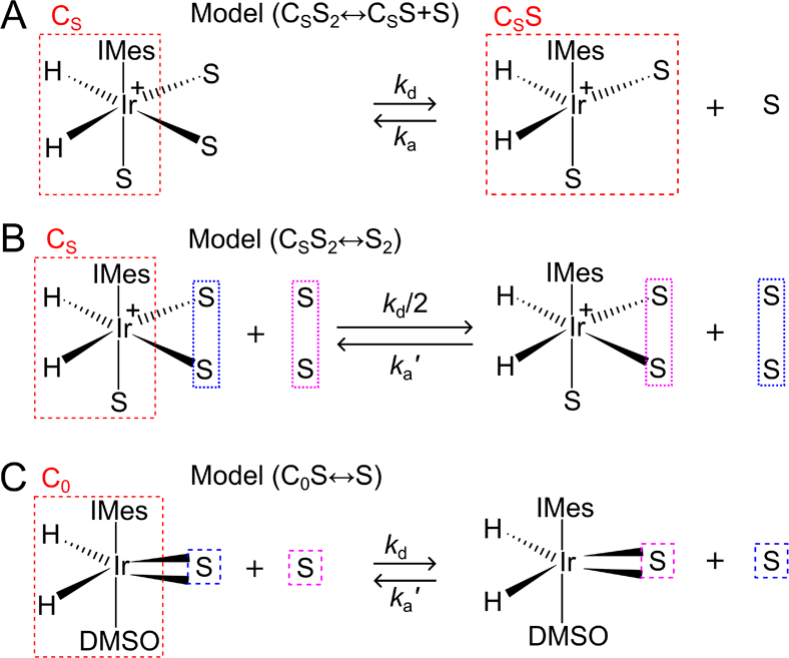
Schematic view of three models used to describe the substrate chemical exchange in SABRE with different degrees of simplification. In model A, C_S_S_2_↔C_S_S+S, while both equatorial substrates can exchange, only one is involved at a time. As a simplification to model A, the C_S_S_2_↔S_2_ model B^23^ assumes that both equatorial substrates exchange simultaneously (with half of the “actual” exchange rate constant). In model C, C_0_S↔S, there is only one substrate, e.g., a bidentate ligand like pyruvate, which now exchanges in one step.^13^

Several approaches could be used to model the observed chemical exchange kinetics of SABRE. In the literature, these vary from the very simple^21,26,33^ to elaborate.^22^ The reaction schemes associated with the models used here are summarized in Figure 3 and employ different levels of complexity.

### SABRE Exchange Model C_S_S_2_↔C_S_S+S

In this case, the SABRE-active iridium complex [Ir(H)_2_(IMes)(substrate)_3_]Cl is represented by C_S_S_2_, where S_2_ is its two equatorial substrate molecules. It is generally assumed that predominantly only equatorial substrate ligands exchange, and this exchange proceeds via a dissociative or S_N_1 type mechanism (Figure 3A).^22^ Thus, one of the two equatorial substrates, S, dissociates, leaving C_S_S as a transient complex. Then, the free substrate can rebind to form the active SABRE complex C_S_S_2_. As such, chemical exchange proceeds in two steps:
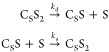
1where *k*_d_ is the monomolecular rate constant of dissociation and *k*_a_ is the bimolecular rate constant of association. In this model, the rates of magnetization exchange between the sites due to cross-relaxation are considered negligible. It should be noted that the concentration of free S in solution impacts the visible back-reaction, which is considered when determining the value of the second-order rate constant *k*_a_.

The chemical kinetics under steady-state equilibrium for each magnetically labeled compound are therefore similar and given by eq 2.
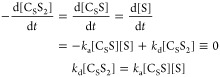
2

In this form 2/*k*_d_ is the lifetime of Ir–S interaction, and *k*_d_/2 is the dissociation rate for the selected one of the two equatorial substrate molecules. At the same time 1/*k*_d_ is the lifetime of active Ir complex (C_S_S_2_). The steady-state concentrations of [S], [C_S_S], and [C_S_S_2_] can be found using eq 2 and equations of mass balance (eq 3):
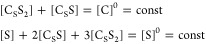
3as
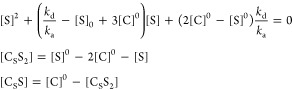
4where [C]^0^ and [S]^0^ are the initial concentrations of Ir precatalyst and substrate.

The evolution of longitudinal magnetization for such a chemical exchange process can be found by solving the Bloch-McConnell equation, which for three compounds can be written in a matrix form as
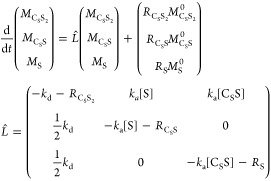
5Here, the equilibrium magnetization *M*_C_S_S_2__^0^ = 2[C_S_S_2_]*P*^0^, *M*_C_S_S_^0^ = [C_S_S]*P*^0^, and *M*_S_^0^ = [S]*P*^0^, the factor 2 in *M*_C_S_S_2__^0^ is to take into account that there are twice as many chemically equivalent spins as in other systems, and *P*^0^ is the thermal polarization of respective spins. Note that here, the production rate of *M*_S_ from *M*_C_S_S_2__ is . The reason is that  because in C_S_S_2_ we add the magnetization of the two constituent substrate molecules (see detailed derivation in SI, eq S1–S4).

Let us introduce the equilibrium constant as  here . Using this approach, we can reduce the number of variables by 1. In addition, because very little is known about putative intermediate C_S_S, which ultimately will be solvated, its relaxation rate can be assumed to be similar to *R*_C_S_S_2__. Under these conditions, *L̂* simplifies as
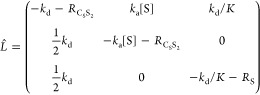
6

Consequently, there are only four unknown parameters: *k*_d_, *k*_a_, *R*_C_S_S_2__, and *R*_S_, while [S] can be calculated with eq 4. Additionally, one can find *R*_S_ from T_1_ relaxation measurements on samples containing only substrate without Ir catalyst. The catalyst should be excluded from this mixture because otherwise, the measured relaxation rate of the free substrate would be effectively averaged between the actual relaxation rates of the free substrate and equatorial substrates. Here, we assumed that the catalyst does not significantly accelerate the relaxation of the free substrate by itself, e.g., it is not paramagnetic, but it changes the relaxation of the substrate when coordinated to the catalyst.

To apply this chemical exchange model, we use the general solution of the Bloch-McConnell equations (eq 5) and the evolution operator in the form given in eq 6. The general solution of this equation is a superposition of exponentially decaying functions with decay rates equal to eigenvalues of *L̂* plus thermal magnetization *M⃗*^0^. The numerical solution of this equation during the mixing time, *d*_mix_, can be found as
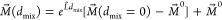
7where *M⃗*(*d*_mix_ = 0) is the initial magnetization of the system at *d*_mix_ = 0 which is, e.g., a result of NMR pulse sequence preparation or spin labeling.

### SABRE exchange model C_S_S_2_↔S_2_

The second model (C_S_S_2_↔S_2_) (Figure 3B) is a simplification of the first C_S_S_2_↔C_S_S+S model. This simplification accelerates the modeling of chemical and spin evolution dynamics in SABRE systems significantly.^23^ Here, again, the active iridium complex C_S_S_2_ has two equatorial substrates. In contrast to model C_S_S_2_↔C_S_S+S, however, both equatorial substrates exchange simultaniously with hypothetical C_S_ and S_2_ species. In the forward reaction, two hyperpolarized (in SABRE experiment) or magnetically encoded (in EXSY or SEXSY experiment) bound substrate molecules are replaced by the S_2_ species. As a result, in a single event, two molecules leave the complex, and thus, we assume that the rate constant for the process is *k*_d_/2. The rate constant of the backward reaction is *k*_a_^’^. It should be noted that these constants do not reflect the actual kinetics of the chemical exchange as the model is a simplification of the more accurate mechanism of exchange reflected by model C_S_S_2_↔C_S_S+S. However, the model is useful for modeling the polarization transfer kinetics between the free and Ir-bound substrate molecule pools. The corresponding chemical exchange reactions are
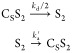
8

The analysis of this model is performed in the similar way to the case of C_S_S_2_↔C_S_S+S model described above. The detailed derivations can be found in SI (eqs S14–S17). Here, we only show the final equation
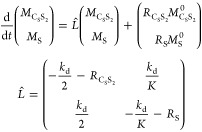
9which is used in this form for fitting (here ).

The revision of the model C_S_S_2_↔S_2_, first discussed by Knecht et al.,^23^ in particular, the introduction of *k*_d_/2 instead of *k*_d_ as a magnetization exchange rate (eq 9) indicates that the previous use of this model needed a more consistent approach in defining what is meant by measured exchange rate constants. For example, when the correction of about 1/2 is applied, the exchange rates obtained with SABRE-INEPT before (SI, Table S9B)^35^ and with SEXSY here (SI, Table S9D) are in good agreement.

### SABRE Exchange Model C_0_S↔S

We also describe a third model (C_0_S↔S) (Figure 3C), which applies to labile bidentate ligands like pyruvate.^13^ This model will also apply to situations where a single substrate molecule binds to the catalyst reversibly, undergoing dissociative ligand loss.^18^ This model is not appropriate for bidentate ligands, which coordinate and can be hyperpolarized within the complex but do not dissociate on the spin relaxation time scale.^45,46^

Here, the active SABRE complex, C_0_S, consists of an Ir core, C_0_, and one substrate molecule, S, which undergoes exchange. Note that in the case of pyruvate, C_0_ would include Ir, IMes, one axial ligand, like DMSO, and two hydride ligands.^13^ When applied to pyruvate, this model will reflect a simplification of the actual mechanism, which is not well understood and likely involves solvent or a second pyruvate coordination ligand to assist in pyruvate exchange. Hence, the rate constants reflect the rates of transmission of pyruvate from bound to free, rather than for a specific mechanistic step. For this model, the two chemical exchange reactions are
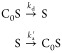
10Again, the similar analysis of this exchange model (see SI, eqs S19–S22) gives the final equation
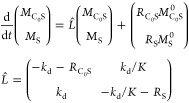
11where .

### Eigenvalues Analysis: Biexponential Fitting

Models C_0_S↔S and C_S_S_2_↔S_2_ can be used to fit the evolution equations as is or after further simplification. Since the general solution to eq 7 is the superposition of biexponential decaying functions with an offset,
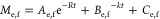
12where e,f stand for equatorial and free substrates, *k* and *R* are minus eigenvalues of *L̂* (eq 9 or eq 11). The eigenvalues of eq 11 (C_0_S↔S) are
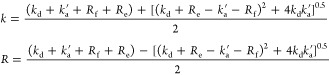
13When relaxation is much slower than chemical exchange (*R*_f_, *R*_e_ ≪ *k*_d_, *k*_a_^’^) these expressions simplify to
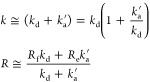
14Therefore, it is tempting to call *k* an effective exchange rate constant (its value will vary with [S]) and *R* an effective relaxation rate of the systems.

From eq 14 and corresponding steady-state equations (eqs S19, S21) one can estimate the rate constants for the model C_0_S↔S:
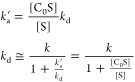
15For the C_S_S_2_↔S_2_ model, one should change  in eqs 13 and 14 and get
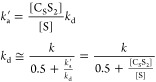
16

### EXSY and SEXSY: Experiments

**Figure 4:**
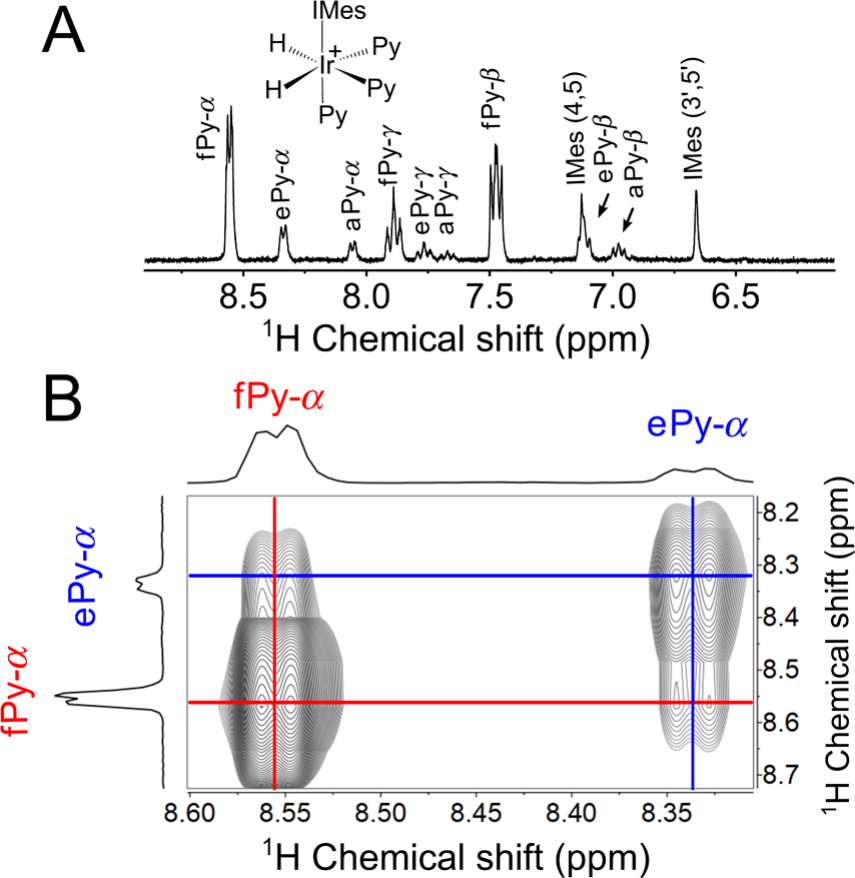
EXSY spectrum. (A) ^1^H NMR spectrum of pyridine (Py) with Ir complex in methanol-*d*_4_ with the assignment of Py peaks (f–free, e–equatorial, a–axial, α, β, and γ protons of Py). (B) 2D EXSY spectrum, which demonstrates the exchange between free (fPy) and bound equatorial (ePy) pyridine molecules measured at *d*_mix_ = 500 ms at 280 K and 7 T using a 2D EXSY sequence. Spectrum on the left was obtained separately using conventional 1D NMR and added here instead of a purely resolved spectrum projection. From (A), one can find the relative ratio  in this experiment.

**Figure 5:**
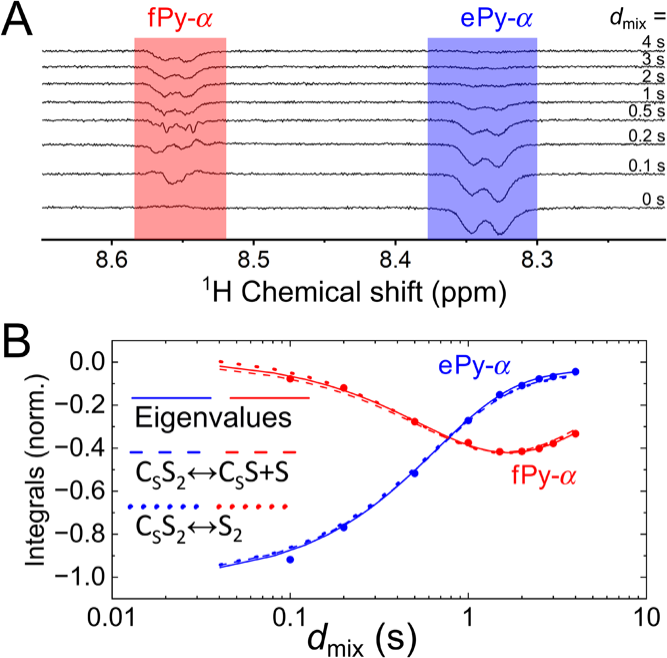
SEXSY spectra. Spectra (A) and fittings of the kinetics of free (fPy-α, red) and equatorial (ePy-α, blue) (B). Signal of Py as a function of mixing time *d*_mix_ at 280 K obtained using 1D SEXSY pulse sequence: fitting with biexponential decay function (solid line, *R* = 0.15 ± 0.02 s^–1^, *k* = 1.55 ± 0.06 s^–1^), fitting with models C_S_S_2_↔C_S_S+S (dashed line, eq 6) and C_S_S_2_↔S_2_ (dotted line, eq 9). Initial concentrations [Ir]^0^ = 4 mM, [Py]^0^ = 40 mM, and experimental estimation of concentrations ratio is  (Figure 4A). The antiphase line shape of fPy-α at short *d*_mix_ results in an overall integral close to zero and does not affect the analysis of the net magnetization exchange.

First, we carried out EXSY (Figure 4) and SEXSY (Figure 5) experiments for the three substrates Py, NAM, and 4AP at different temperatures. Note that we also attempted to perform similar measurements for acetonitrile and metronidazole (both are common SABRE compounds^47,48^); however, evaluation of the exchange rates was not possible because the chemical shifts of the protons of the bound equatorial substrate (eS) and the free substrate (fS) were too close together. For each compound, exchange rates were determined at five temperatures 280, 283, 288, 293, and 298 K; all corresponding data are available in SI.

In EXSY, when a sufficiently long *d*_mix_ (e.g., 500 ms for Py at 280 K) was employed, the cross peaks between bound and free substrates were clearly visible (Figure 4B) and indicative of the corresponding chemical exchange process. In SEXSY, at *d*_mix_ = 0 s, the bound substrate is inverted while the signal for the free substrate is greatly suppressed by phase cycling (Figure 5A). Increasing the time delay between the last two pulses allows one to follow the transfer of magnetization from bound to free Py. After about 1.5 s, magnetization on the free form reaches its maximum and then goes down (Figure 5B).

In Table 1, all estimated exchange rates using these experimental data are given, and in the following sections we will discuss the implications of using different exchange models on the resulting exchange rate values. More fitting parameters, such as *k*_a_^’^ and *R*_f_ relaxation of the bound substrate are given in Table S3–S4. In the same tables, one can find that we also performed the same simulations with free *R*_f_ parameter, which proved that using *R*_f_ values based on experimental *T*_1_ data (Table S2) improved our fitting.

**Table 1:** Modeled Dissociation Exchange Rate Constants Using Two Sets of Experiments (SEXSY and EXSY) with Two Models (C_S_S_2_↔C_S_S+S and C_S_S_2_↔S_2_) for Three Substrates (Py, 4AP, and NAM)^a^

			Model C_S_S_2_↔C_S_S+S	Model C_S_S_2_↔S_2_		Model C_S_S_2_↔S_2_: Eigenvalues analysis (biexponential fitting)			
			SEXSY	EXSY	SEXSY	SEXSY			
Substrate	*T* (K)	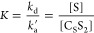	*k*_d_ (s^–1^)	*k*_d_ (s^–1^)	*k*_d_ (s^–1^) (eq 9)	*k* (s^–1^)	*R* (s^–1^)	*k*_d_ (s^–1^) from *k*	Mean *k*_d_ (s^–1^)
Py	280	7.46	1.9 ± 0.1	1.1 ± 0.1	1.7 ± 0.1	1.55 ± 0.06	0.15 ± 0.02	2.44 ± 0.10	1.81 ± 0.04
	283	8.61	3.4 ± 0.1	1.7 ± 0.2	3.0 ± 0.1	2.57 ± 0.20	0.10 ± 0.06	4.17 ± 0.33	3.01 ± 0.08
	288	9.72	7.6 ± 0.2	3.6 ± 0.5	6.4 ± 0.3	4.64 ± 0.22	0.08 ± 0.03	7.69 ± 0.37	6.99 ± 0.15
	293	15.82	14.9 ± 0.9	6.0 ± 0.8	10.5 ± 0.8	7.95 ± 0.56	0.18 ± 0.06	14.12 ± 0.99	10.92 ± 0.43
	298	11.04	37.4 ± 3.9	25.3 ± 2.9	30.2 ± 3.0	17.05 ± 0.96	0.13 ± 0.02	28.88 ± 1.62	29.31 ± 1.21
4AP	280	21.86	0.80 ± 0.04	0.7 ± 0.1	0.90 ± 0.04	0.82 ± 0.01	0.08 ± 0.005	1.51 ± 0.03	1.13 ± 0.02
	283	20.83	1.4 ± 0.1	1.0 ± 0.1	1.7 ± 0.1	1.09 ± 0.03	0.09 ± 0.005	1.99 ± 0.05	1.68 ± 0.04
	288	22.48	3.6 ± 0.1	1.7 ± 0.2	3.6 ± 0.1	2.00 ± 0.09	0.09 ± 0.005	3.68 ± 0.16	3.38 ± 0.07
	293	23.85	5.5 ± 0.6	3.5 ± 0.4	8.2 ± 2.8	2.90 ± 0.45	0.10 ± 0.019	5.36 ± 0.82	4.36 ± 0.30
	298	24.33	7.3 ± 0.8	8.0 ± 0.9	20.1 ± 10.5	5.63 ± 0.46	0.07 ± 0.005	10.41 ± 0.85	8.65 ± 0.48
NAM	280	23.48	1.5 ± 0.1	0.6 ± 0.1	1.3 ± 0.1	1.20 ± 0.06	0.16 ± 0.015	2.22 ± 0.11	1.38 ± 0.04
	283	24.25	2.1 ± 0.1	0.95 ± 0.11	2.1 ± 0.1	1.62 ± 0.09	0.15 ± 0.013	2.99 ± 0.16	1.93 ± 0.05
	288	24.92	3.5 ± 0.2	2.4 ± 0.3	5.8 ± 0.6	1.73 ± 0.09	0.16 ± 0.008	3.20 ± 0.16	3.29 ± 0.10
	293	26.37	6.3 ± 0.4	5.7 ± 0.6	9.9 ± 0.8	4.06 ± 0.33	0.11 ± 0.011	7.55 ± 0.62	6.84 ± 0.29
	298	27.22	8.0 ± 0.8	12.5 ± 1.3	24.8 ± 9.8	5.13 ± 0.68	0.099 ± 0.008	9.55 ± 1.27	9.24 ± 0.58

a Error intervals are standard deviation values for the given variables obtained using MATLAB nonlinear regression function “nlinfit”. Error intervals in model C_S_S_2_↔S_2_ applied to EXSY experiments were calculated using eq. 32 of ref (44). The last column is weighted mean *k*_d_ and the combined standard deviation calculated from four *k*_d_ values considering their error intervals.

### SEXSY: Simulations

A straightforward way to estimate an exchange rate is to fit the intensity of signals from free (*M*_f_) and bound (*M*_e_) substrates using a biexponential decay; eq 12. It is reasonable to assume that the faster rate corresponds to effective chemical exchange because it increases the signal intensity of the free substrate while the slower rate is effective relaxation (Figure 5B). Using eq 16 and the ratio of integrals obtained from the regular ^1^H spectra (Figure 4A, Table 1) we can estimate the values of *k*_d_ and *k*_a_^’^.

Using eq 5 for the model C_S_S_2_↔C_S_S+S and eq S17 for the model C_S_S_2_↔S_2_, one can also fit the same data using the concentration ratio as a restraint on *k*_a_^’^ and leaving *k*_d_ and relaxation parameters as the fit variables. Also, by measuring *T*_1_ of the free substrate, we improved our fitting by reducing the number of relaxation rate constants used in the fitting (Table S2–S3). The needed concentrations for the model C_S_S_2_↔C_S_S+S can be calculated using initial concentrations, e.g., for Py [Ir]^0^ = 4 mM, [Py]^0^ = 40 mM. As an initial condition for eq 7, we assumed that only C_S_S_2_ was initially polarized and inverted as a result of phase cycling in SEXSY experiment; other molecules are not polarized.

Finally, using the three fitting procedures, for Py at 280 K, we obtained *k*_d_ = 2.4 ± 0.1 s^–1^ using the biexponential fit together with the model C_S_S_2_↔S_2_, 1.7 ± 0.1 s^–1^ when directly fitted the SEXSY kinetics using model C_S_S_2_↔S_2_ (eq 9) and 1.9 ± 0.1 s^–1^ when the C_S_S_2_↔C_S_S+S model was fitted. The data for each molecule and approach are listed in Table 1, corresponding fits are in the SI and exemplary fits are shown in Figure 5B. As one can see, similar values can be obtained using three approaches and two models. The fitted dissociation rates of model C_S_S_2_↔S_2_ are generally lower by 10–20% than those provided by C_S_S_2_↔C_S_S+S model.

The equilibrium constants *K* for 4AP and NAM were approximately two times higher than that for Py, and the main reason is that we used the doubled amount of substrate.

At higher temperatures of 293 K and especially 298 K, exchange rates were so fast that even for *d*_mix_ ≈ 0 s (90° selective pulse was 40 ms), we had significant antiphase polarization on the free substrate. This factor limits our fitting model’s robustness and increases uncertainties, indicating that at higher temperatures, one should use rapid broad-band pulse sequences like SABRE-INEPT^35^ or EXSY. Luckily, the antiphase type of spectrum at short *d*_mix_ has made minimal contributions to the analysis of net magnetization, which we have pursued here.

In our simulations, we did not consider any intermolecular NOE polarization transfer effects as we did not see any hints of this.

### EXSY: Simulations

There is a significant difference between 2D EXSY and SEXSY. In the case of SEXSY, complete exchange kinetics is measured and analyzed with the corresponding exchange model. Even visual analysis can be useful in checking the quality of the fit. In the case of EXSY, however, the measurement of complete exchange kinetics is extremely time-consuming, so in practice, only one point in the linear slope of the kinetics is measured, which leads to additional biases. Another disadvantage of the EXSY approach is the partial overlap of the signals, making the integration less accurate. To describe the EXSY experiment, we cannot use the solution for eq 7 directly since we have to calculate magnetization at different time points and the relative ratio between diagonal and cross peaks (Figure 4).

Here, we used a simple A↔B exchange model to describe EXSY (eqs S23–S33, SI)^44,49^ and adapted it to our simplified exchange model C_S_S_2_↔S_2_ (eqs S35–S37, SI). The result is our derivations allow us to estimate the exchange constants as
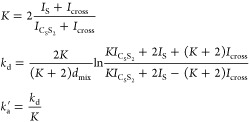
17

Here *I* is the corresponding integral in 2D spectrum for equatorially bound (*I*_C_S_S_2__) or free (*I*_S_) substrates, or average integral of cross peaks (*I*_cross_) after mixing time *d*_mix_. To obtain such simple equations, we needed an assumption of equal relaxation rates for spins in the complex and the free substrate. This is certainly not correct for the case of SABRE.^50^ Still, it allows a significant decrease in the required experimental time as it is enough to measure only a single EXSY spectrum at a given temperature to obtain the exchange rates. A more precise implementation of EXSY is to measure two EXSY spectra with *d*_mix_ ≠ 0 and *d*_mix_ = 0, respectively. This allows one to account for the difference in the spin–lattice relaxation rates. Because the second reference spectrum should be acquired for each temperature, this accurate analysis doubles the required measurement time. This is impractical and raises the risks of data irreproducibility because the long-term stability of SABRE samples is not guaranteed. We performed some preliminary tests with Py, which showed that despite the assumption of equal spin–lattice relaxation rates, both approaches provide very similar rate constants (Table S5). Therefore, we used the approach based on the single-spectrum acquisition and the resultant eq 17.

Another problem with EXSY measurements is the fact that the estimated exchange rate constant depends on the mixing time used for spectra acquisition. In particular, a series of EXSY measurements with *d*_mix_ varied from 20 to 100 ms were performed for the Py at 298 K (Table S6). It was found that estimated *k*_d_ grows with *d*_mix_, reaching the plateau at *d*_mix_ ≥ 50 ms. However, it is possible that the dependence can be more complex in different experimental conditions or in different mixing time ranges. Therefore, it can be concluded that, in general, the EXSY approach is less reliable for measuring exchange rates in SABRE compared to SEXSY.

### Enthalpy Δ*H*^‡^ and Entropy Δ*S*^‡^ of Activation

**Figure 6:**
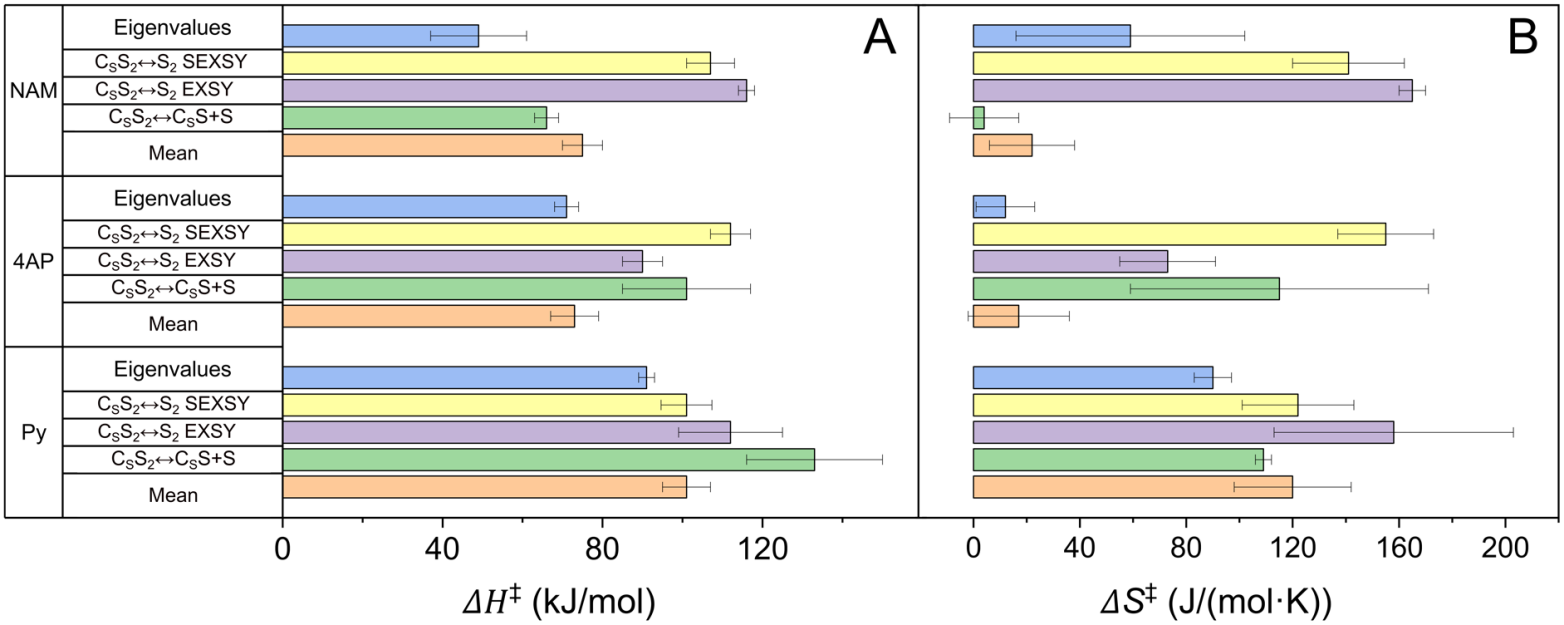
Enthalpies Δ*H*^‡^ (A) and entropies Δ*S*^‡^ (B) of activation for IrIMes-derived complexes with Py, 4AP and NAM as a substrate. Δ*H*^‡^ and Δ*S*^‡^ values were obtained from dissociation exchange constants measured with the model C_S_S_2_↔C_S_S+S (SEXSY, green), with the model C_S_S_2_↔S_2_ (EXSY, purple), with the model C_S_S_2_↔S_2_ (SEXSY, yellow), eigenvalues analysis (blue) and the values calculated using mean *k*_d_ (orange). The values are obtained by fitting *k*_d_ (Table 1, Figure S7, SI) and are given in Table S8, SI.

By using the dissociation rates *k*_d_ (Table 1), we were able to calculate activation enthalpies Δ*H*^‡^ and entropies Δ*S*^‡^ (Table S8, Figure 6) using the Eyring equation (eq S40, SI). Enthalpies of activation were between 36 and 120 kJ/mol and entropies of activation were between 7 and 180 J/(mol·K) for all complexes.

## Conclusions

The EXSY sequence is generally more time-consuming and less robust than the SEXSY sequence. Lower spectral resolution in indirect dimension in EXSY reduces the precision of spectral integration. However, it has benefits at higher temperatures where rapid chemical exchange occurs during the selective pulse of SEXSY. Therefore, SEXSY is preferable and more accurate than EXSY for a relatively slow exchange compared to the selective excitation pulse duration. Both SEXSY and EXSY sequences could not be applied to acetonitrile and metronidazole (data not shown here) as the substrate ^1^H chemical shifts almost do not change upon association with IrIMes. In such cases, or when deuterium labeling is used to prolong relaxation, one should use heteronuclei labeling and EXSY, spin order transfer, or a combination of both.^35^

Using two different exchange models, three approaches for fitting SEXSY and one approach for fitting EXSY experiments, we obtained comparable exchange rates. We demonstrated a connection between the complete SABRE model C_S_S_2_↔C_S_S+S and the reduced model C_S_S_2_↔S_2_ often used for the polarization transfer simulations.^23^ As a result, we identified some new limitations for using the C_S_S_2_↔S_2_ model: effective lifetime of the complex and mass balance conditions cannot be immediately satisfied. An ideal solution for spin-dynamic simulations would be to use the model C_S_S_2_↔C_S_S+S; however, such an approach is slow because this model is inevitably nonlinear, and we do not have sufficient information on the elusive intermediate C_S_S. An important aspect is that for bidentate ligands like pyruvate^13^ the exchange model C_0_S↔S, the often-used SABRE model, should work without any identified restraints. Hence, it can be used to optimize spin order transfer to pyruvate, which was already utilized for in vivo imaging.^41,42^

## Data Availability

Corresponding raw data and simulation Matlab scripts can be accessed via Zenodo DOI: 10.5281/zenodo.11073108.

## Supporting Information Available

Additional details on formal analysis and comparison of exchange models, analysis of 2D EXSY data fitting, additional experimental details, biexponential fitting of EXSY data, results of SEXSY fitting, EXSY protocols validation, enthalpies and entropies of activation (PDF)
