# Supplementary material for: Modeling Ligand Exchange Kinetics in Iridium Complexes Catalyzing SABRE Nuclear Spin Hyperpolarization
Source: Anal Chem. 2024 Jul 8;96(29):11790–9. doi: 10.1021/acs.analchem.4c01374 (PMC11270526; doi:10.1021/acs.analchem.4c01374)
Supplement: Supplementary file 1 — ac4c01374_si_001.pdf [file ac4c01374_si_001.pdf]

Supporting Information for:

Modeling ligand exchange kinetics in iridium complexes catalyzing SABRE nuclear spin hyperpolarization

Oleg G. Salnikov[a]*†, Charbel D. Assaf[b]†, Anna P. Yi[a,c], Simon B. Duckett[d], Eduard Y. Chekmenev[e], Jan-Bernd Hövener[b], Igor V. Koptyug[a], Andrey N. Pravdivtsev[b]*

[a] International Tomography Center SB RAS, 3A Institutskaya St., 630090 Novosibirsk, Russia

[b] Section Biomedical Imaging, Molecular Imaging North Competence Center (MOIN CC), Department of Radiology and Neuroradiology, University Medical Center Kiel, Kiel University, Am Botanischen Garten 14, 24118, Kiel, Germany

[c] Novosibirsk State University, 2 Pirogova St., 630090 Novosibirsk, Russia

[d] Centre for Hyperpolarization in Magnetic Resonance (CHyM), University of York, Heslington YO10 5NY, UK, E-mail: simon.duckett@york.ac.uk

[e] Department of Chemistry, Integrative Biosciences (Ibio), Karmanos Cancer Institute (KCI), Wayne State University, Detroit, MI 48202, United States

* salnikov@tomo.nsc.ru; andrey.pravdivtsev@rad.uni-kiel.de

† These authors contributed equally

Contents

Chemical exchange models S-3

The most rigorous substrate exchange SABRE model (C_SS_2↔C_SS+S) S-3

Simplification of the first substrate exchange model (C_SS_2↔S) S-5

C_SS_2↔S_2 model S-7

C_0S↔S model S-8

Comparison of magnetization redistribution in three exchange SABRE models S-9

Fitting of 2D EXSY S-11

A simple A↔B (C_0S↔S or C_SS_2↔S_2) case S-11

Fitting of exchange rates as a function of temperature S-14

Methods S-15

Spectral acquisition parameters S-15

Data processing S-15

1D SEXSY NMR analysis S-15

2D EXSY NMR analysis S-15

Experimental data S-17

Fitting of SEXSY measurements S-20

C_SS_2↔C_SS+S model fitting for SEXSY experimental data S-20

C_SS_2↔S_2 model fitting for SEXSY experimental data S-21

EXSY protocols validation S-23
EXSY protocols experimental comparison S-23
EXSY measurements at variable mixing time S-23
Optimal mixing time for EXSY S-24
Enthalpies and entropies of activation S-25
Comparison with literature exchange rates S-26
References S-27

Chemical exchange models

According to the literature and our observations only equatorial substrate S exchanges with the free substrate; the exchange of the axial substrate is negligible. Below we give additional hints that explain how to analyze the SABRE kinetics.

The most rigorous substrate exchange SABRE model ($C_SS_2 \leftrightarrow C_SS+S$)

The model is detailed in the main text (Eq. 1-7) and here we will detail only some derivations.

Using Eq. 2 on chemical exchange, one also can derive the evolution of magnetization. The following approach is based on the Bloch-McConnell equation. The magnetization is proportional to concentration and polarization and can be defined for the three compounds:

$$\begin{cases} M_{\mathrm{C_SS_2}} = [\mathrm{C_SS_2}]\left(P_{\mathrm{C_SS_2^a}} + P_{\mathrm{C_SS_2^b}}\right) \\ M_{\mathrm{C_SS}} = [\mathrm{C_SS}]P_{\mathrm{C_SS}} \\ M_{\mathrm{S}} = [\mathrm{S}]P_{\mathrm{S}} \end{cases} \qquad \text{(Eq. S1)}$$

$\mathrm{C_SS_2}$ consists of two chemically equivalent coordinated substrate molecules indicated with superscripts as $\mathrm{C_SS_2^a}$ and $\mathrm{C_SS_2^b}$, while $\mathrm{C_SS}$ and S consist of only one substrate molecule in each species which involved in the chemical exchange. Note that both $\mathrm{C_SS_2}$ and $\mathrm{C_SS}$ have one axial nonexchangable substrate. As a result, there are two constituents in the magnetization of $\mathrm{C_SS_2}$ (Eq. S1). The corresponding thermal equilibrium magnetization $M^0_{\mathrm{C_SS_2}}$, $M^0_{\mathrm{C_SS}}$ or M^0_{S} can be obtained by substituting the polarization values with the thermal polarization P^0.

Then, the corresponding kinetic equation for magnetization of $\mathrm{C_SS_2}$ is as follows:

$$\frac{\mathrm{d}M_{\mathrm{C_SS_2}}}{dt} = -k_{\mathrm{d}}[\mathrm{C_SS_2}]\left(P_{\mathrm{C_SS_2^a}} + P_{\mathrm{C_SS_2^b}}\right) + k_{\mathrm{a}}[\mathrm{C_SS}][\mathrm{S}]\left(P_{\mathrm{C_SS}} + P_{\mathrm{S}}\right) - R_{\mathrm{C_SS_2}}\left(M_{\mathrm{C_SS_2}} - M^0_{\mathrm{C_SS_2}}\right) \qquad \text{(Eq. S2)}$$

Here, the first term reflects the fact that due to the dissociation event only one of two substrates can dissociate and the other one goes to $\mathrm{C_SS}$ state, the second term shows that the $M_{\mathrm{C_SS_2}}$ can be combined from $\mathrm{C_SS}$ and S with polarization values of $P_{\mathrm{C_SS}}$ and P_{S} respectively and the last term is relaxation of $M_{\mathrm{C_SS_2}}$ to thermal equilibrium. Hereafter, R_{X} is the relaxation rate of species X. It is easy to check the validity of the equation when you put the polarization of all compounds to be the same and equal to polarization at thermal equilibrium. Then this equation becomes equivalent to Eq. 2 of the main text.

The second term of Eq. S2 can be simplified and written in terms of corresponding magnetizations of exchanging species as $k_{\mathrm{a}}P_{\mathrm{C_SS}}[\mathrm{C_SS}][\mathrm{S}] + k_{\mathrm{a}}[\mathrm{C_SS}]P_{\mathrm{S}}[\mathrm{S}] = k_{\mathrm{a}}\left([\mathrm{S}]M_{\mathrm{C_SS}} + [\mathrm{C_SS}]M_{\mathrm{S}}\right)$.

In the same fashion let's derive equations for another magnetization kinetics:

$$\begin{aligned} \frac{\mathrm{d}M_{\mathrm{C_SS}}}{\mathrm{d}t} &= +k_{\mathrm{d}}[\mathrm{C_SS_2}]\frac{\left(P_{\mathrm{C_SS_2^a}} + P_{\mathrm{C_SS_2^b}}\right)}{2} - k_{\mathrm{a}}[\mathrm{C_SS}][\mathrm{S}]P_{\mathrm{C_SS}} - R_{\mathrm{C_SS}}\left(M_{\mathrm{C_SS}} - M^0_{\mathrm{C_SS}}\right) \\ \frac{\mathrm{d}M_{\mathrm{S}}}{\mathrm{d}t} &= +k_{\mathrm{d}}[\mathrm{C_SS_2}]\frac{\left(P_{\mathrm{C_SS_2^a}} + P_{\mathrm{C_SS_2^b}}\right)}{2} - k_{\mathrm{a}}[\mathrm{C_SS}][\mathrm{S}]P_{\mathrm{S}} - R_{\mathrm{S}}\left(M_{\mathrm{S}} - M^0_{\mathrm{S}}\right) \end{aligned} \qquad \text{(Eq. S3)}$$

Here all terms are similar to those in Eq. S2 except for the first one. Here $\mathrm{C_SS}$ will get polarization of $P_{\mathrm{C_SS_2^a}}$ or polarization of $P_{\mathrm{C_SS_2^b}}$ with 50% probability, so the factor of ½ should be introduced. And now, we can combine Eqs. S1-S3 and obtain the evolution of magnetization equations for all three species:

$$\begin{cases} \frac{\mathrm{d}M_{\mathrm{C_SS_2}}}{\mathrm{d}t} = -k_\mathrm{d}M_{\mathrm{C_SS_2}} + k_\mathrm{a}[\mathrm{S}]M_{\mathrm{C_SS}} + k_\mathrm{a}[\mathrm{C_SS}]M_\mathrm{S} - R_{\mathrm{C_SS_2}}\left(M_{\mathrm{C_SS_2}} - M^0_{\mathrm{C_SS_2}}\right) \\ \frac{\mathrm{d}M_{\mathrm{C_SS}}}{\mathrm{d}t} = +\frac{1}{2}k_\mathrm{d}M_{\mathrm{C_SS_2}} - k_\mathrm{a}[\mathrm{S}]M_{\mathrm{C_SS}} - R_{\mathrm{C_SS}}\left(M_{\mathrm{C_SS}} - M^0_{\mathrm{C_SS}}\right) \\ \frac{\mathrm{d}M_\mathrm{S}}{\mathrm{d}t} = +\frac{1}{2}k_\mathrm{d}M_{\mathrm{C_SS_2}} - k_\mathrm{a}[\mathrm{C_SS}]M_\mathrm{S} - R_\mathrm{S}\left(M_\mathrm{S} - M^0_\mathrm{S}\right) \end{cases} \quad \text{(Eq. S4)}$$

In the matrix form this equation is given in Eq. 5 in the main text.

Simplification of the first substrate exchange model (C_SS_2↔S)

The first model can be further simplified. We start with Eqs. S2 and S3 and definition in Eq. S1, but this time let's also add up $\frac{dM_{C_SS}}{dt}$ and $\frac{dM_S}{dt}$:

$$\begin{cases} \frac{dM_{C_SS_2}}{dt} = -k_d M_{C_SS_2} + k_a'\left(\frac{[S]}{[C_SS]}M_{C_SS} + M_S\right) - R_{C_SS_2}\left(M_{C_SS_2} - M_{C_SS_2}^0\right) \\ \frac{d\left(M_S + M_{C_SS}\right)}{dt} = +k_d M_{C_SS_2} - k_a'\left(\frac{[S]}{[C_SS]}M_{C_SS} + M_S\right) - R_{C_SS}\left(M_{C_SS} - M_{C_SS}^0\right) - R_S\left(M_S - M_S^0\right) \end{cases} \quad \text{(Eq. S5)}$$

where $k_a' = k_a[C_SS]$. Because of relatively rapid exchange and the fact that C_SS has been never observed experimentally so far, let's set that $M_S = \frac{[S]}{[C_SS]}M_{C_SS}$. This is for sure correct at thermal equilibrium and when exchange is much faster than relaxation. This gives us

$$\begin{cases} \frac{dM_{C_SS_2}}{dt} = -k_d M_{C_SS_2} + k_a' 2M_S - R_{C_SS_2}\left(M_{C_SS_2} - M_{C_SS_2}^0\right) \\ \frac{d\left(M_S + M_{C_SS}\right)}{dt} \cong \left(1 + \frac{[C_SS]}{[S]}\right)\frac{dM_S}{dt} \cong +k_d M_{C_SS_2} - k_a' 2M_S - \left(\frac{R_{C_SS}[C_SS]}{[S]} + R_S\right)\left(M_S - M_S^0\right) \end{cases} \quad \text{(Eq. S6)}$$

As $\frac{[C_SS]}{[S]} \ll 1$, we can set it equal to 0, and if R_{C_SS} is not so much different from R_S we finally obtain the evolution of magnetization as

$$\begin{cases} \frac{dM_{C_SS_2}}{dt} = -k_d M_{C_SS_2} + 2k_a' M_S - R_{C_SS_2}\left(M_{C_SS_2} - M_{C_SS_2}^0\right) \\ \frac{dM_S}{dt} \cong +k_d M_{C_SS_2} - 2k_a' M_S - R_S\left(M_S - M_S^0\right) \end{cases} \quad \text{(Eq. S7)}$$

One can see that our error in this simplification is as small as $\frac{[C_SS]}{[S]}$ which we can estimate after the simulation. In the matrix form Eq. S7 is written as

$$\frac{d}{dt}\begin{pmatrix} M_{C_SS_2} \\ M_S \end{pmatrix} = \hat{L}\begin{pmatrix} M_{C_SS_2} \\ M_S \end{pmatrix} + \begin{pmatrix} R_{C_SS_2} M_{C_SS_2}^0 \\ R_S M_S^0 \end{pmatrix}$$
$$\hat{L} = \begin{pmatrix} -k_d - R_{C_SS_2} & 2k_a' \\ k_d & -2k_a' - R_S \end{pmatrix} \quad \text{(Eq. S8)}$$

This equation is easy to diagonalize, therefore in the main text we compared eigenvalue analysis and direct fitting using a general solution of Eq. 7 applied to fitting 1D SEXSY and 2D EXSY experiments.

It can be noticed that Eq. S8 can be obtained if one considers the following kinetic scheme

$$C_SS_2 \xrightarrow{k_d} S$$
$$S \xrightarrow{2k_a' = 2k_a[C_SS]} C_SS_2 \quad \text{(Eq. S9)}$$

This scheme can be rationalized as a simplified scheme of C_SS_2↔ C_SS+S model in case when C_SS complex is neglected (this is reasonable considering that it has not been observed experimentally so far and, thus, has short lifetime and very small concentration compared to other species and especially to [S]). Hence, here the SABRE exchange is treated as a pseudo-first order process, and the model is designated as C_SS_2↔S.

The pseudo first order association rate constant was set as $2k_a[C_SS]$ in order to obtain correct equations for magnetization (see Eq. S6). From Eq. S9, the chemical kinetics and steady-state concentrations can be found as

$$-\frac{d[C_SS_2]}{dt} = \frac{d[S]}{dt} = -2k_a'[S] + k_d[C_SS_2]$$
$$k_d[C_SS_2] = 2k_a'[S] \quad \text{(Eq. S10)}$$

If again, $K = \frac{[\mathrm{S}]}{[\mathrm{C_S S_2}]}$ then $k_\mathrm{a}^{'} = \frac{k_\mathrm{d}}{2K}$. Using the steady state concentrations and mass balance equation

$$[\mathrm{C_S S_2}] \cong [\mathrm{C}]^0 = \mathrm{const}$$
$$[\mathrm{S}] + 3[\mathrm{C_S S_2}] \cong [\mathrm{S}] + 3[\mathrm{C}]^0 \cong [\mathrm{S}]^0 = \mathrm{const} \qquad \text{(Eq. S11)}$$

Hence $[\mathrm{S}] \cong [\mathrm{S}]^0 - 3[\mathrm{C}]^0$ and $\frac{[\mathrm{C_S S_2}]}{[\mathrm{S}]} = \frac{1}{[\mathrm{S}]^0/[\mathrm{C}]^0 - 3}$. The $[\mathrm{S}]$ value is known from the initial concentrations of substrate and Ir complex, while the $\frac{[\mathrm{C_S S_2}]}{[\mathrm{S}]}$ ratio can be measured experimentally from an ^{1}H spectrum.

This model has only 3 fitting parameters as k_a' is obtained from the k_d, concentrations of substrate and catalyst. Also, it is clear that this equation is very similar to Eq. 12 of the main text except that here all rate constants are 2 times higher than in Eq. 12. The problem with Eq. S9 is that two substrate molecules of $\mathrm{C_S S_2}$ are formally converted to only one free substrate molecule which cannot have the same magnetization. Still it is very useful to find simplification that leads to such 2×2 matrix as it results in analytical insights into the system discussed below.

For the eigenvalues analysis for this model one should change above k_a' with $2k_\mathrm{a}'$ and use different equation of mass-balance than for the model $\mathrm{C_0S}$↔S:

$$k \cong (k_\mathrm{d} + 2k_\mathrm{a}') = k_\mathrm{d}\left(1 + \frac{2k_\mathrm{a}'}{k_\mathrm{d}}\right)$$
$$R \cong \frac{R_\mathrm{f} k_\mathrm{d} + 2R_\mathrm{e} k_\mathrm{a}'}{k_\mathrm{d} + 2k_\mathrm{a}'}$$
$$k_\mathrm{a}' = \frac{1}{2}\frac{[\mathrm{C_S S_2}]}{[\mathrm{S}]} k_\mathrm{d} \qquad \text{(Eq. S12)}$$
$$k_\mathrm{d} \cong \frac{k}{1 + \frac{2k_\mathrm{a}'}{k_\mathrm{d}}} = \frac{k}{1 + \frac{[\mathrm{C_S S_2}]}{[\mathrm{S}]}}$$

Interesting that using eigenvalue analysis for both models $\mathrm{C_S S_2}$↔$\mathrm{S_2}$ and $\mathrm{C_S S_2}$↔S resulting k_d is about the same ($\frac{[\mathrm{C_S S_2}]}{[\mathrm{S}]} \ll 1$) while k_a' is about 2 times different.

$C_SS_2 \leftrightarrow S_2$ model

The model $C_SS_2 \leftrightarrow S_2$ is introduced with the following chemical exchange reactions:

$$\begin{gathered}\mathrm{C_SS_2} \xrightarrow{k_\mathrm{d}/2} \mathrm{S_2} \\ \mathrm{S_2} \xrightarrow{k'_\mathrm{a}} \mathrm{C_SS_2}\end{gathered} \quad \text{(Eq. S13)}$$

The chemical kinetics and steady-state concentrations can be found analogously to Eq. 2

$$\begin{gathered}-\frac{\mathrm{d}[\mathrm{C_SS_2}]}{\mathrm{d}t} = \frac{\mathrm{d}[\mathrm{S_2}]}{\mathrm{d}t} = -k'_\mathrm{a}[\mathrm{S_2}] + \frac{k_\mathrm{d}}{2}[\mathrm{C_SS_2}] \\ \frac{k_\mathrm{d}}{2}[\mathrm{C_SS_2}] = k'_\mathrm{a}[\mathrm{S_2}] = \frac{k'_\mathrm{a}}{2}[\mathrm{S}]\end{gathered} \quad \text{(Eq. S14)}$$

where k'_a is a pseudo first-order association rate constant and $[\mathrm{S_2}] = [\mathrm{S}]/2$, where $[\mathrm{S}]$ is concentration of free substrate molecules and again $K = \frac{k_\mathrm{d}}{k'_\mathrm{a}} = \frac{[\mathrm{S}]}{[\mathrm{C_SS_2}]}$. Let us write the mass balance equations as

$$\begin{gathered}[\mathrm{C_SS_2}] = [\mathrm{C}]^0 = \mathrm{const} \\ 2[\mathrm{S_2}] + 3[\mathrm{C_SS_2}] = [\mathrm{S}]^0 = \mathrm{const}\end{gathered} \quad \text{(Eq. S15)}$$

Here it is assumed that all Ir is in the form of C_SS_2. If this model is to be correct $[\mathrm{C_SS}] \ll [\mathrm{C_SS_2}], [\mathrm{S}]$, so the intermediate $\mathrm{C_SS}$ can be neglected and the two models ultimately converge to the same result (see comparison in **Figures S1** and **S2**). Below, we will compare how well they fit experimental data.

Next, one can find the concentrations of constituents as

$$\begin{gathered}[\mathrm{S}] = \frac{[\mathrm{S}]^0}{1+3\frac{k'_\mathrm{a}}{k_\mathrm{d}}} = 2[\mathrm{S_2}] \\ [\mathrm{C_SS_2}] = \frac{k'_\mathrm{a}}{k_\mathrm{d}}[\mathrm{S}]\end{gathered} \quad \text{(Eq. S16)}$$

The corresponding evolution of longitudinal magnetization can be found as

$$\begin{gathered}\frac{\mathrm{d}}{\mathrm{d}t}\begin{pmatrix} M_{\mathrm{C_SS_2}} \\ M_{\mathrm{S_2}} \end{pmatrix} = \hat{L}\begin{pmatrix} M_{\mathrm{C_SS_2}} \\ M_{\mathrm{S_2}} \end{pmatrix} + \begin{pmatrix} R_{\mathrm{C_SS_2}} M^0_{\mathrm{C_SS_2}} \\ R_{\mathrm{S_2}} M^0_{\mathrm{S_2}} \end{pmatrix} \\ \hat{L} = \begin{pmatrix} -\frac{k_\mathrm{d}}{2} - R_{\mathrm{C_SS_2}} & k'_\mathrm{a} \\ \frac{k_\mathrm{d}}{2} & -k'_\mathrm{a} - R_{\mathrm{S_2}} \end{pmatrix}\end{gathered} \quad \text{(Eq. S17)}$$

During fitting, $M_{\mathrm{C_SS_2}}$ should link to the integral of bound substrate and M_S which equals $M_{\mathrm{S_2}}$ – to the free substrate. The latter equality is correct since $[\mathrm{S}] = 2[\mathrm{S_2}]$ and the number of spins in $\mathrm{S_2}$ is twice the number of magnetically labeled spins in S. As K which equals $\frac{[\mathrm{S}]}{[\mathrm{C_SS_2}]}$ can be obtained from the experiment, it is possible to substitute $k'_\mathrm{a} = \frac{k_\mathrm{d}}{K}$. Then, Eq. S17 can be rewritten as Eq. 9 (see the main text) and used in this form for fitting.

$C_0S \leftrightarrow S$ model

This model $C_0S \leftrightarrow S$ is introduced as

$$\mathrm{C_0S} \overset{k_\mathrm{d}}{\rightarrow} \mathrm{S} \qquad \text{(Eq. S18)}$$
$$\mathrm{S} \overset{k'_\mathrm{a}}{\rightarrow} \mathrm{C_0S}$$

The governing equation on the concentrations of the constituents is as follows:

$$-\frac{\mathrm{d}[\mathrm{C_0S}]}{\mathrm{d}t} = \frac{\mathrm{d}[\mathrm{S}]}{\mathrm{d}t} = -k'_\mathrm{a}[\mathrm{S}] + k_\mathrm{d}[\mathrm{C_0S}] \qquad \text{(Eq. S19)}$$
$$k_\mathrm{d}[\mathrm{C_0S}] = k'_\mathrm{a}[\mathrm{S}]$$

again $K = \frac{k_\mathrm{d}}{k'_\mathrm{a}} = \frac{[\mathrm{S}]}{[\mathrm{C_0S}]}$. From the steady state concentrations and equation of mass balance (assuming that all Ir is in the form of C_0S)

$$[\mathrm{C_0S}] = [\mathrm{C_0}]^0 = \mathrm{const} \qquad \text{(Eq. S20)}$$
$$[\mathrm{S}] + [\mathrm{C_0S}] = [\mathrm{S}]^0 = \mathrm{const}$$

one can find the concentrations of constituents as

$$[\mathrm{S}] = \frac{[\mathrm{S}]^0}{1+\frac{k'_\mathrm{a}}{k_\mathrm{d}}} \qquad \text{(Eq. S21)}$$
$$[\mathrm{C_0S}] = \frac{k'_\mathrm{a}}{k_\mathrm{d}}[\mathrm{S}]$$

Here, we wrote the mass balance for the case of bidentate ligand, while for the case of co-substrate the mass balance is different. As $K = \frac{[\mathrm{S}]}{[\mathrm{C_0S}]}$ can be calculated or obtained from the experiment, we can also substitute $k'_\mathrm{a} = \frac{k_\mathrm{d}}{K}$.

Magnetization kinetics can be derived for C_0S and S resulting in the system:

$$\begin{cases} \frac{\mathrm{d}M_{\mathrm{C_0S}}}{\mathrm{d}t} = -k_\mathrm{d}M_{\mathrm{C_0S}} + k'_\mathrm{a}M_\mathrm{S} - R_{\mathrm{C_0S}}\left(M_{\mathrm{C_0S}} - M^0_{\mathrm{C_0S}}\right) \\ \frac{\mathrm{d}M_\mathrm{S}}{\mathrm{d}t} = k_\mathrm{d}M_{\mathrm{C_0S}} - k'_\mathrm{a}M_\mathrm{S} - R_\mathrm{S}\left(M_\mathrm{S} - M^0_\mathrm{S}\right) \end{cases} \qquad \text{(Eq. S22)}$$

In the matrix form it is written in Eq. 11 of the main text.

Comparison of magnetization redistribution in three exchange SABRE models

To demonstrate the validity of models and their difference, we plotted evolution of magnetization components for three models $C_SS_2 \leftrightarrow C_SS+S$, $C_SS_2 \leftrightarrow S_2$ and $C_SS_2 \leftrightarrow S$ using the same k_a' and k_d, and initial concentrations for free substrate and catalyst (**Figure S1**). The results are identical for $C_SS_2 \leftrightarrow C_SS+S$ and $C_SS_2 \leftrightarrow S_2$ models while different for $C_SS_2 \leftrightarrow S$. We found that if one changes $k_d \rightarrow k_d/2$ and $K \rightarrow K/2$ in the $C_SS_2 \leftrightarrow S$ model then the results will be the same as for the two other models (which indeed should be like this considering the 2-fold difference in rate constants between $C_SS_2 \leftrightarrow S_2$ and $C_SS_2 \leftrightarrow S$ models, see above).

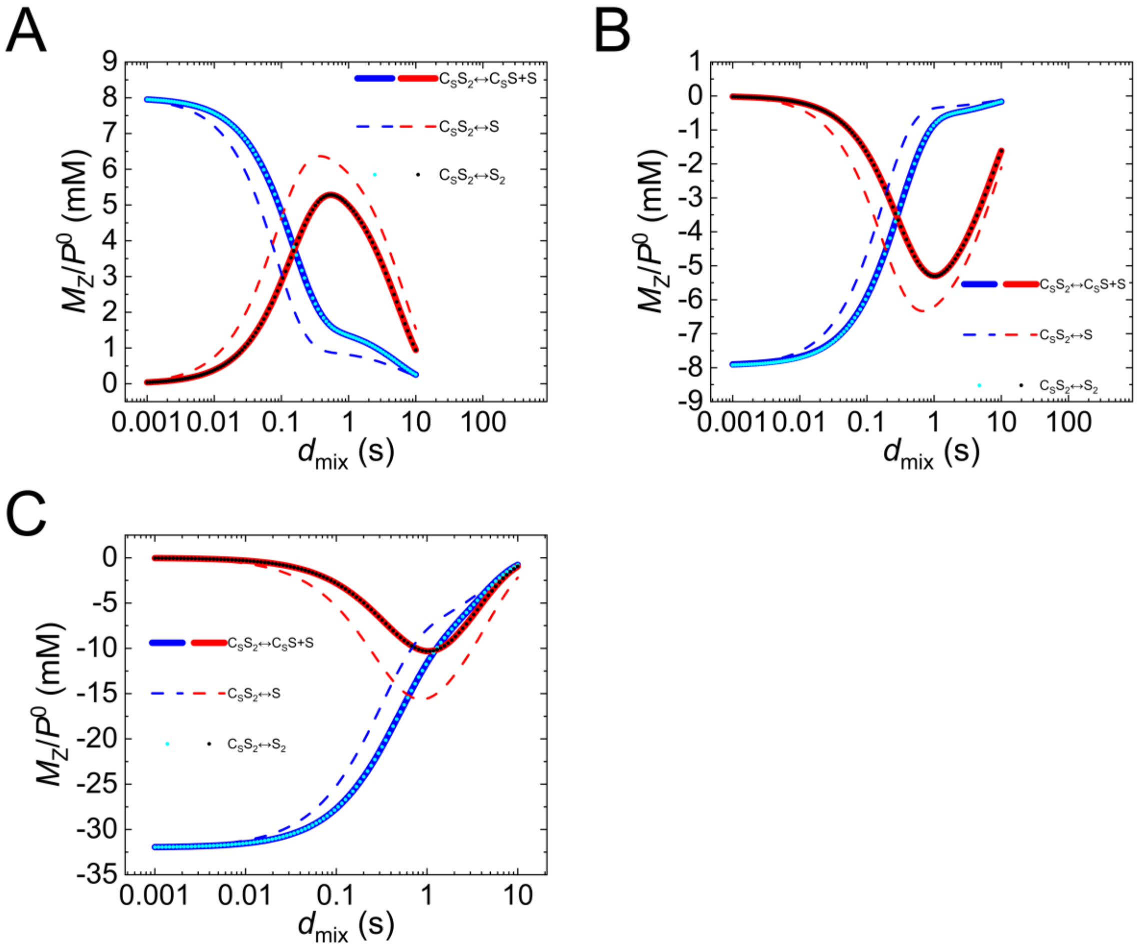

Figure S1. Simulations of magnetization evolution for three models: $C_SS_2 \leftrightarrow C_SS+S$ (lines), $C_SS_2 \leftrightarrow S_2$ (dotted line), $C_SS_2 \leftrightarrow S$ (dashed line)) at three different conditions.

(A) k_d = 10 s^{-1}, k_a = 1000 (mM·s)$^{-1}$ [C]^0 = 4 mM, [S]^0=40 mM, from this we calculated equilibrium concentrations and exchange constant: [S] = 28 mM, $[C_SS]$ = 0.0014 mM and $[C_SS_2]$ = 3.9986 mM, and initial magnetization values in terms of its thermal equilibrium magnetization are $M_S(d_{mix} = 0) = M_{C_SS}(d_{mix} = 0) = 0$, $M_{C_SS_2}(d_{mix} = 0) = 2M^0_{C_SS_2}$.

(B) k_d = 5 s^{-1}, k_a = 10 (mM·s)$^{-1}$ [C]^0 = 4 mM, [S]^0=80 mM, from this we calculated equilibrium concentrations and exchange constant: [S] = 68.0292 mM, $[C_SS]$ = 0.0292 mM and $[C_SS_2]$ = 3.9702 mM, and initial magnetization values in terms of its thermal equilibrium magnetization are $M_S(d_{mix} = 0) = M_{C_SS}(d_{mix} = 0) = 0$, $M_{C_SS_2}(d_{mix} = 0) = -2M^0_{C_SS_2}$.

(C) k_d = 2 s^{-1}, k_a = 1000 (mM·s)$^{-1}$ [C]^0 = 16 mM, [S]^0=80 mM, from this we calculated equilibrium concentrations and exchange constant: [S] = 32 mM, $[C_SS]$ = 0.0001 mM and $[C_SS_2]$ = 15.999 mM,

and initial magnetization values in terms of its thermal equilibrium magnetization are $M_{\mathrm{S}}(d_{\mathrm{mix}}=0)=M_{\mathrm{C_SS}}(d_{\mathrm{mix}}=0)=0$, $M_{\mathrm{C_SS_2}}(d_{\mathrm{mix}}=0)=-2M^0_{\mathrm{C_SS_2}}$.

In these simulations, we considered that $\vec{M}^0$= 0 in Eq 7, as if we have phase cycling and by considering thermal magnetization is constant.

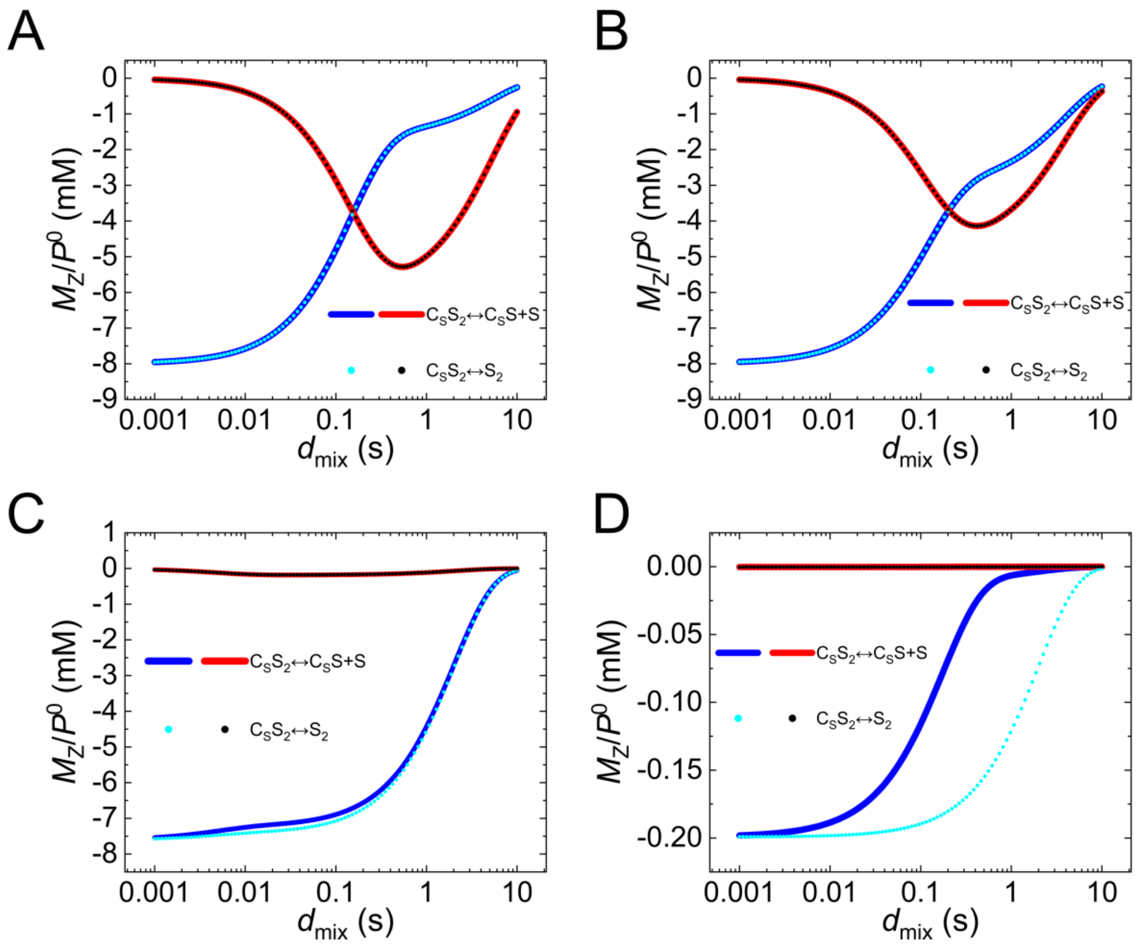

Figure S2: Simulations of magnetization evolution for three models: $C_SS_2 \leftrightarrow C_SS+S$ (lines), $C_SS_2 \leftrightarrow S_2$ (dotted line) at different substrate initial concentrations. For all simulations, k_{d} = 10 s^{-1}, k_{a} = 1000 $(mM \cdot s)^{-1}$, and $[C]^0$ = 4 mM were used. (A) $[S]^0$= 40 mM, with calculated $[C_SS]$ = 0.0014 mM (B) $[S]^0$= 24 mM, with calculated $[C_SS]$ = 0.0033 mM (C) $[S]^0$= 12 mM, with calculated $[C_SS]$ = 0.1951 mM and (D) $[S]^0$= 8.1 mM, with calculated $[C_SS]$ = 3.9003 mM. Calculated $[C_SS]$ is only for model $C_SS_2 \leftrightarrow C_SS+S$.

Fitting of 2D EXSY

A simple A↔B (C_0S↔S or C_SS_2↔S_2) case

The matrix of the EXSY peaks integrals is calculated using matrix exponent in the following way

$$\hat{I}(d_{\mathrm{mix}}) = e^{\hat{L}d_{\mathrm{mix}}}\hat{M}^0 \qquad \text{(Eq. S23)}$$

where $\hat{L}$ is an exchange matrix, d_{mix} is mixing time and $\hat{M}^0$ is a diagonal matrix of equilibrium magnetizations.

Let us first consider a simple kinetic model of two-site exchange between the two sites A and B:

$$\mathrm{A} \xrightarrow{k_{\mathrm{d}}} \mathrm{B} \qquad \mathrm{B} \xrightarrow{k_{\mathrm{a}}} \mathrm{A} \qquad \text{(Eq. S24)}$$

where k_{a} and k_{d} are the first order exchange rate constant, R_{A} and R_{B} are the corresponding spin-lattice relaxation rates. Here, the rates of magnetization exchange between the A and B sites due to cross-relaxation are considered negligible. This scheme gives the following exchange matrix $\hat{L}$:

$$\hat{L} = \begin{pmatrix} -k_{\mathrm{d}} - R_{\mathrm{A}} & k_{\mathrm{a}} \\ k_{\mathrm{d}} & -k_{\mathrm{a}} - R_{\mathrm{B}} \end{pmatrix} \qquad \text{(Eq. S25)}$$

while

$$\hat{M}^0 = \begin{pmatrix} M_{\mathrm{A}}^0 & 0 \\ 0 & M_{\mathrm{B}}^0 \end{pmatrix}, \quad \hat{I}(d_{\mathrm{mix}}) = \begin{pmatrix} I_{\mathrm{A}}(d_{\mathrm{mix}}) & I_{\mathrm{B\to A}}(d_{\mathrm{mix}}) \\ I_{\mathrm{A\to B}}(d_{\mathrm{mix}}) & I_{\mathrm{B}}(d_{\mathrm{mix}}) \end{pmatrix} \qquad \text{(Eq. S26)}$$

The solution of the obtained equation is well-known and can be found elsewhere[1]:

$$\begin{aligned} I_{\mathrm{A}}(d_{\mathrm{mix}}) &= \tfrac{1}{2}\left[\left(1-\tfrac{\delta}{D}\right) + \left(1+\tfrac{\delta}{D}\right)e^{-2Dd_{\mathrm{mix}}}\right]e^{-(\sigma-D)d_{\mathrm{mix}}}M_{\mathrm{A}}^0 \\ I_{\mathrm{B}}(d_{\mathrm{mix}}) &= \tfrac{1}{2}\left[\left(1+\tfrac{\delta}{D}\right) + \left(1-\tfrac{\delta}{D}\right)e^{-2Dd_{\mathrm{mix}}}\right]e^{-(\sigma-D)d_{\mathrm{mix}}}M_{\mathrm{B}}^0 \\ I_{\mathrm{cross}}(d_{\mathrm{mix}}) = I_{\mathrm{A\to B}}(d_{\mathrm{mix}}) = I_{\mathrm{B\to A}}(d_{\mathrm{mix}}) &= \tfrac{1}{2}\left[1-e^{-2Dd_{\mathrm{mix}}}\right]e^{-(\sigma-D)d_{\mathrm{mix}}}\bar{M} \end{aligned} \qquad \text{(Eq. S27)}$$

where $D = \sqrt{\delta^2 + k_{\mathrm{a}}k_{\mathrm{d}}}$, $\delta = \frac{1}{2}(k_{\mathrm{d}} - k_{\mathrm{a}} + R_{\mathrm{A}} - R_{\mathrm{B}})$, $\sigma = \frac{1}{2}(k_{\mathrm{a}} + k_{\mathrm{d}} + R_{\mathrm{A}} + R_{\mathrm{B}})$, $\bar{M} = M_{\mathrm{B}}^0\frac{k_{\mathrm{a}}}{D} = M_{\mathrm{A}}^0\frac{k_{\mathrm{d}}}{D}$. In practice one should set $I_{\mathrm{cross}}(d_{\mathrm{mix}}) = \frac{1}{2}\left(I_{\mathrm{A\to B}}(d_{\mathrm{mix}}) + I_{\mathrm{B\to A}}(d_{\mathrm{mix}})\right)$ in order to compensate for experimental errors.

Let us introduce the equilibrium constant

$$K = \frac{k_{\mathrm{d}}}{k_{\mathrm{a}}} = \frac{[\mathrm{B}]}{[\mathrm{A}]} = \frac{M_{\mathrm{B}}^0}{M_{\mathrm{A}}^0} \qquad \text{(Eq. S28)}$$

Next, let us assume that the spin-lattice relaxation rates are the same for the two exchanging positions ($R = R_{\mathrm{A}} = R_{\mathrm{B}}$). This assumption is certainly not correct for the SABRE system[2] but still it is a common way of EXSY analysis. More correct approach involves acquisition of two EXSY NMR spectra: the first one with non-zero d_{mix} and the second one with $d_{\mathrm{mix}} = 0$. This allows one to account for the difference in the spin-lattice relaxation rates. However, the preliminary tests with pyridine substrate showed that both approaches provide very similar rate constants. As acquisition of two EXSY spectra effectively doubles the required time, we used the approach based on the single-spectrum acquisition and the math presented in this section (with the assumption of $R = R_{\mathrm{A}} = R_{\mathrm{B}}$). The assumption of equal spin-lattice relaxation rates gives

$$\delta = \frac{1}{2}(k_\text{d} - k_\text{a}) = \frac{K-1}{2}k_\text{a}$$
$$D = \sqrt{\delta^2 + k_\text{a}k_\text{d}} = \sqrt{\frac{K^2-2K+1}{4}{k_\text{a}}^2 + {k_\text{a}}^2 K} = \frac{1}{2}(1+K)k_\text{a}$$
$$\frac{\delta}{D} = \frac{K-1}{K+1}$$
$$\bar{M} = \frac{2}{K+1}M_\text{B}^0 \qquad \text{(Eq. S29)}$$
$$\sigma = \frac{1}{2}(1+K)k_\text{a} + R$$
$$\sigma - D = R$$
$$1 + \frac{\delta}{D} = \frac{2K}{K+1}$$
$$1 - \frac{\delta}{D} = \frac{2}{K+1}$$

Substituting the Eq. S29 into Eq. S27 gives

$$I_\text{A}(d_\text{mix}) = \frac{1}{K(K+1)}\left[1 + Ke^{-(K+1)k_\text{a}d_\text{mix}}\right]e^{-Rd_\text{mix}}M_\text{B}^0$$
$$I_\text{B}(d_\text{mix}) = \frac{1}{K+1}\left[K + e^{-(K+1)k_\text{a}d_\text{mix}}\right]e^{-Rd_\text{mix}}M_\text{B}^0 \qquad \text{(Eq. S30)}$$
$$I_\text{cross}(d_\text{mix}) = \frac{1}{K+1}\left[1 - e^{-(K+1)k_\text{a}d_\text{mix}}\right]e^{-Rd_\text{mix}}M_\text{B}^0$$

Next, it can be easily shown that

$$KI_\text{A}(d_\text{mix}) - I_\text{B}(d_\text{mix}) = \frac{1-K}{K+1}\left[1 - e^{-(K+1)k_\text{a}d_\text{mix}}\right]e^{-Rd_\text{mix}}M_\text{B}^0$$
$$\frac{KI_\text{A} - I_\text{B}}{I_{cross}} = 1 - K \qquad \text{(Eq. S31)}$$
$$K = \frac{I_\text{B} + I_\text{cross}}{I_\text{A} + I_\text{cross}}$$

Similarly,

$$KI_\text{A}(d_\text{mix}) + I_\text{B}(d_\text{mix}) = \left[1 + e^{-(K+1)k_\text{a}d_\text{mix}}\right]e^{-Rd_\text{mix}}M_\text{B}^0$$
$$\frac{KI_\text{A} + I_\text{B}}{I_\text{cross}} = \frac{1 + e^{-(K+1)k_\text{a}d_\text{mix}}}{1 - e^{-(K+1)k_\text{a}d_\text{mix}}}(K+1) \qquad \text{(Eq. S32)}$$

Eq. S32 can be easily solved to give rate constants

$$k_\text{d} = \frac{K}{(K+1)d_\text{mix}}\ln\frac{KI_\text{A} + I_\text{B} + (K+1)I_\text{cross}}{KI_\text{A} + I_\text{B} - (K+1)I_\text{cross}} \qquad \text{(Eq. S33)}$$
$$k_\text{a} = \frac{k_\text{d}}{K}$$

All these equations are immediately applied to **$C_0S \leftrightarrow S$** model of exchange. One should set

$$\text{A} \rightarrow \text{C}_0\text{S}$$
$$\text{B} \rightarrow \text{S}$$
$$k_\text{a} \rightarrow k'_\text{a} = k_\text{a}[\text{C}_0] \qquad \text{(Eq. S34)}$$
$$K = \frac{k_\text{d}}{k'_\text{a}} = \frac{[\text{S}]}{[\text{C}_0\text{S}]}$$

This equation can also be immediately applied to **$C_SS_2 \leftrightarrow S_2$** model with following changes. If we set $k'_\text{d} = \frac{k_\text{d}}{2}$, then matrix and steady-state equation are

$$\hat{L} = \begin{pmatrix} -k'_\text{d} - R_\text{A} & k'_\text{a} \\ k'_\text{d} & -k'_\text{a} - R_\text{B} \end{pmatrix} \qquad \text{(Eq. S35)}$$
$$k'_\text{d}[\text{C}_\text{S}\text{S}_2] = k'_\text{a}[\text{S}_2]$$

Here, we can introduce new effective $K' = \frac{k'_\text{d}}{k'_\text{a}} = \frac{[\text{S}_2]}{[\text{C}_\text{S}\text{S}_2]}$ and equations will coincide with the $\text{A} \leftrightarrow \text{B}$ model. Hence one can use the solution written above as $\text{A} \rightarrow \text{C}_\text{S}\text{S}_2$, $\text{B} \rightarrow \text{S}_2 \rightarrow \text{S}$,

$$K' = \frac{k'_\mathrm{d}}{k'_\mathrm{a}} = \frac{k_\mathrm{d}}{2k'_\mathrm{a}} = \frac{K}{2} = \frac{I_{\mathrm{S}_2} + I_\mathrm{cross}}{I_{\mathrm{C_S}S_2} + I_\mathrm{cross}}$$

$$k'_\mathrm{d} = \frac{K'}{(K'+1)d_\mathrm{mix}} \ln \frac{K' I_{\mathrm{C_S S_2}} + I_{\mathrm{S}_2} + (K'+1) I_\mathrm{cross}}{K' I_{\mathrm{C_S S_2}} + I_{\mathrm{S}_2} - (K'+1) I_\mathrm{cross}} \qquad \text{(Eq. S36)}$$

$$k'_\mathrm{a} = k'_\mathrm{d}/K'$$

And finally, for normal (not temporary introduced values) it is

$$K = 2\frac{I_\mathrm{S} + I_\mathrm{cross}}{I_{\mathrm{C_S}S_2} + I_\mathrm{cross}}$$

$$k_\mathrm{d} = \frac{2K}{(K+2)d_\mathrm{mix}} \ln \frac{K I_{\mathrm{C_S S_2}} + 2I_\mathrm{S} + (K+2) I_\mathrm{cross}}{K I_{\mathrm{C_S S_2}} + 2I_\mathrm{S} - (K+2) I_\mathrm{cross}} \qquad \text{(Eq. S37)}$$

$$k'_\mathrm{a} = \frac{k_\mathrm{d}}{K}$$

Here, we also substituted S_2 with S as integrals are identical because $M_\mathrm{S} = M_{\mathrm{S}_2}$.

Fitting of exchange rates as a function of temperature

Arrhenius's equation has the following form

$$k_\mathrm{d} = Ae^{-\frac{E_\mathrm{a}}{RT}} \qquad \text{(Eq. S38)}$$

Which is convenient to fit with a linear function as follows:

$$\ln(k_\mathrm{d}) = \ln(A) - \frac{E_\mathrm{a}}{RT} \qquad \text{(Eq. S39)}$$

Here k_d is in s^{-1}.

We also estimated the entropy and enthalpy of activation by using the Eyring equation in the form

$$k_\mathrm{d} = \frac{k_\mathrm{B}T}{h}\exp\left(\frac{\Delta S^\ddagger}{R} - \frac{\Delta H^\ddagger}{RT}\right) \qquad \text{(Eq. S40)}$$

Which is convenient to fit with a linear function as follows:

$$\mathrm{R}\ln\left(\frac{k_\mathrm{d}\cdot h}{k_\mathrm{B}\cdot T}\right) = -\Delta H^\ddagger\left(\frac{1}{T}\right) + \Delta S^\ddagger \qquad \text{(Eq. S41)}$$

Methods

Spectral acquisition parameters

The acquisition parameters for 1D SEXSY NMR spectra were as follows: ns = 8, ds = 4, d1 = 12 s. Mixing time d_{mix} was varied to measure the kinetic curves.

The acquisition parameters for 1D regular NMR spectra were as follows ns = 8, ds = 0, d1 = 40 s.

The acquisition parameters for 2D EXSY NMR spectra were as follows: td2 = 4096, td1 = 128, ns = 8, ds = 16, d1 = 3 s.

The acquisition parameters for T1ir NMR spectra were as follows: td2 = 16, ns = 1, ds = 0, d1 was at least 6 times greater than the measured T_1 values.

Data processing

All spectra were analyzed using spectral data analyzing software Bruker TopSpin (4.0.7), Bruker Dynamics Center (2.5.5), MestReNova (14.2.2), and Origin (2021). Data were modelled using Origin, MATLAB (R2021a), Microsoft Office Excel 2007. Data were simulated using MOIN spin-library.

1D SEXSY NMR analysis

The spectra were phase-corrected and baseline-corrected, and the signals of free and equatorial-bound protons (α-protons for Py, H-5 protons for NAM, and β-protons for 4AP, **Figure S2**) were integrated. Chemical shifts of these protons in the free and bound forms are given in Table SX. Next, the obtained kinetic curves were global fitted using Eq. 12. The obtained K values as well as $\frac{[S]}{[C_S S_2]}$ ratio measured from thermal ^{1}H NMR spectra were used for the calculations of rate constants according to Eq. 16.

For more precise fitting, we need to have T_1 relaxation rate constant of the free substrate (**Table S2**). For this purpose, similar sample but without catalyst was prepared and T_1 was measured using inversion-recovery pulse sequence.

2D EXSY NMR analysis

The following Fourier transform parameters were used for the processing of 2D EXSY NMR spectra: si2 = si1 = 2048, wdw2 = wdw1 = QSINE, ssb2 = ssb1 = 2. The spectra were phase-corrected and baseline-corrected. The diagonal peaks and cross peaks corresponding to the signals of free and equatorial-bound protons (α-protons for Py, H-5 protons for NAM, and β-protons for 4AP) were integrated using rectangular area selection mode. The obtained integrals were employed for the calculation of substrate dissociation rate constants using Eq. S37.

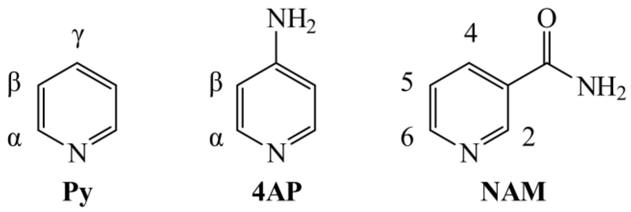

Figure S3. Chemical structures of pyruvate (Py), 4-aminopyridine (4AP), nicotinamide (NAM), and proton numbering.

Table S1. ^{1}H Chemical shifts for the axial, bound and free substrate for Py, 4AP, NAM at 283 K.

Substrate - ^{1}H Chemical shift	Axial substrate (ppm)	Equatorial substrate (ppm)	Free substrate (ppm)
Pyridine (Py) – α protons	8.05	8.33	8.55
4-aminopyridine (4AP) – β-protons	6.03	6.16	6.56
Nicotinamide (NAM) – H-5 protons	7.05	7.34	7.63

Table S2. T_1 measurements for all substrates at different temperatures.

Substrate – protons	Temperature (K)	T_1 (s)
Pyridine (Py) – α protons	280	10.8 ± 0.7
	283	11.7 ± 0.9
	288	16.6 ± 1.0
	293	21.2 ± 0.6
	298	24.6 ± 0.3
4-aminopyridine (4AP) – β-protons	280	6.5 ± 0.1
	283	6.85 ± 0.08
	288	7.8 ± 0.1
	293	9.01 ± 0.09
	298	10.2 ± 0.09
Nicotinamide (NAM) – H-5 protons	280	4.0 ± 0.2
	283	4.3 ± 0.2
	288	4.8 ± 0.2
	293	5.4 ± 0.2
	298	6.25 ± 0.11

Experimental data

ALL measured SEXSY curves together with biexponential fitting are given below in Figures S3-S5.

The resulting biexponential fitting parameters *R* and *k* are given in Table 1.

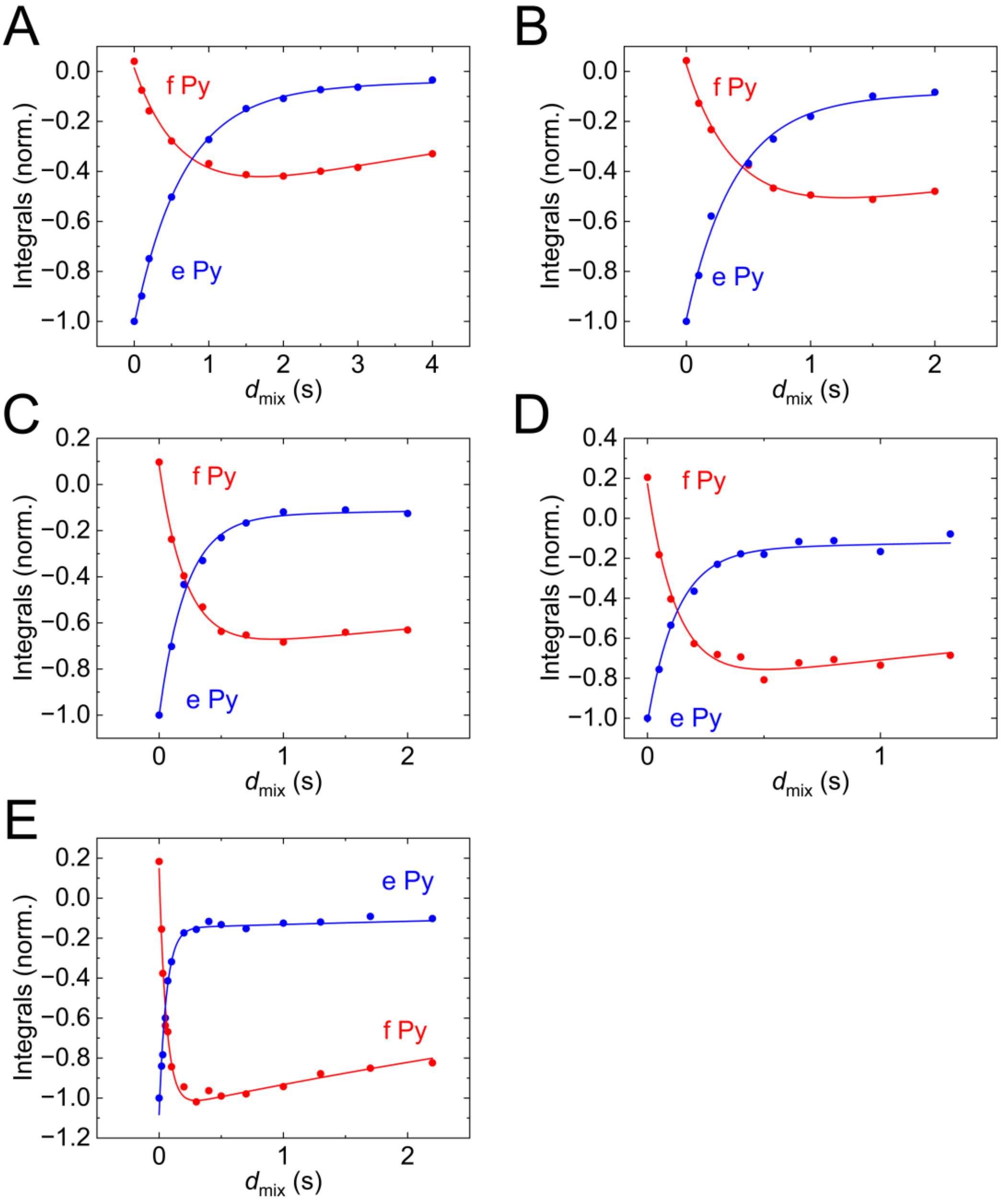

Figure S4. 1D SEXSY kinetic curves for pyridine at different temperatures: (A) 7 °C, (B) 10 °C, (C) 15 °C, (D) 20 °C, (E) 25 °C.

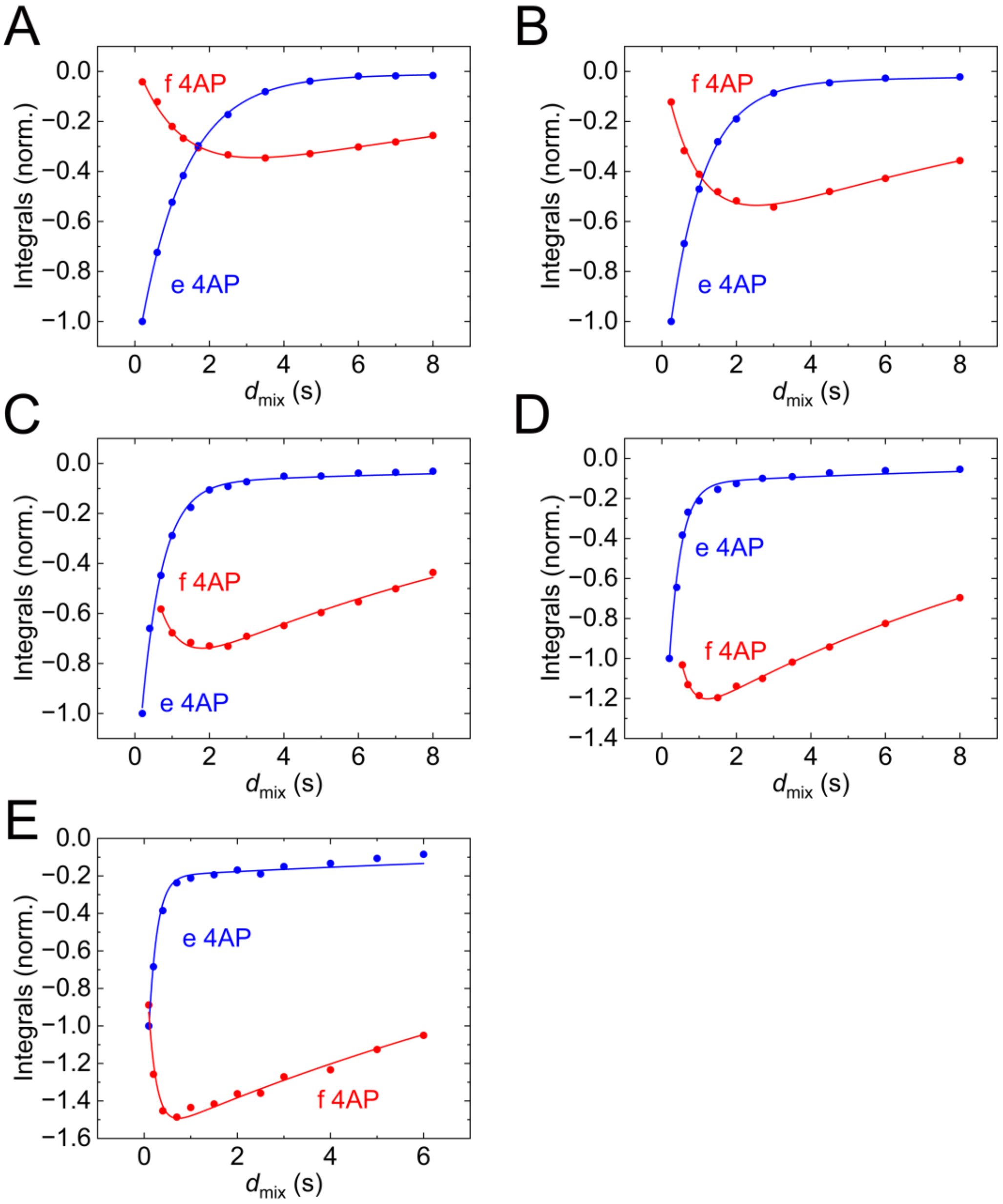

Figure S5. 1D SEXSY kinetic curves for 4-aminopyridine at different temperatures: (A) 7 °C, (B) 10 °C, (C) 15 °C, (D) 20 °C, (E) 25 °C.

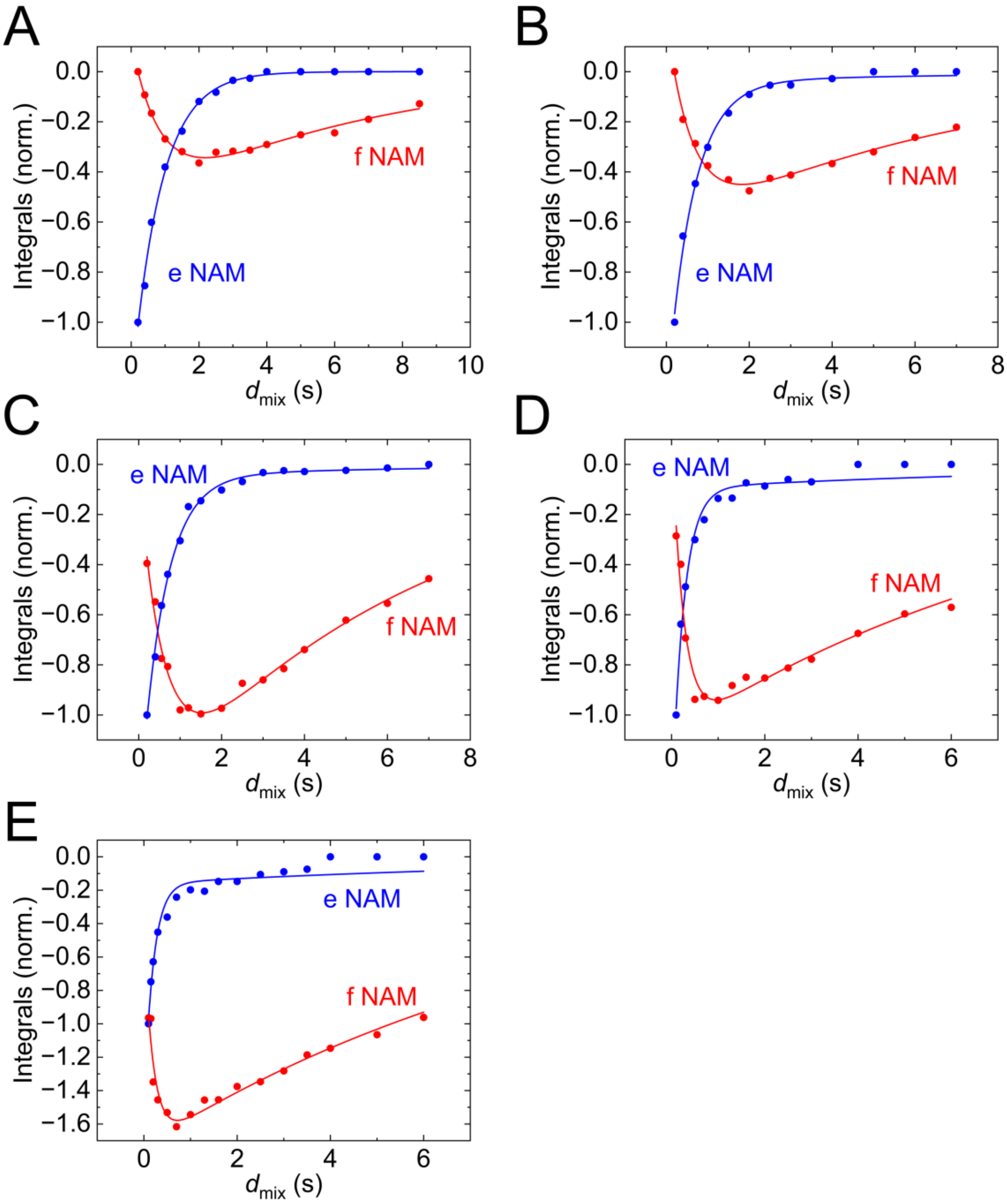

Figure S6. 1D SEXSY kinetic curves for nicotinamide at different temperatures: (A) 7 °C, (B) 10 °C, (C) 15 °C, (D) 20 °C, (E) 25 °C.

Fitting of SEXSY measurements

$C_SS_2 \leftrightarrow C_SS+S$ model fitting for SEXSY experimental data

We fitted SEXSY experimental data using $C_SS_2 \leftrightarrow C_SS+S$ model. Two studies were made to perform the best fitting: first, we do normal fit with variable relaxation constant rates, and second by using fixed relaxation constant rates based on T_1 measurement of the free substrate at different temperatures. $R_f = 1/T_1$ (**Table S2**) is the relaxation constant rate for the free substrate in this model and R_e is the relaxation rate of the bound substrate in this model.

Table S3. Fitting parameters for all substrates using model $C_SS_2 \leftrightarrow C_SS+S$ for SEXSY experimental data.

Substrate	T (K)	$\frac{[S]}{[C_SS_2]}$	*Model* $C_SS_2 \leftrightarrow C_SS+S$, (with variable R_f values)				*Model* $C_SS_2 \leftrightarrow C_SS+S$, (with fixed R_f values)			
			k_d (s^{-1})	$k_{a'}$ (s^{-1})	R_f (s^{-1})	R_e (s^{-1})	k_d (s^{-1})	$k_{a'}$ (s^{-1})	R_f (s^{-1})	R_e (s^{-1})
Py	280	7.46	1.87 ± 0.06	0.25 ± 0.01	0.07 ± 0.00	0.55 ± 0.02	1.92 ± 0.07	0.26 ± 0.01	0.0926 ± 0.006	0.51 ± 0.02
	283	8.61	3.31 ± 0.17	0.38 ± 0.02	0.06 ± 0.06	0.61 ± 0.10	3.36 ± 0.13	0.39 ± 0.01	0.0855 ± 0.007	0.56 ± 0.05
	288	9.72	7.55 ± 0.21	0.78 ± 0.02	0.05 ± 0.04	0.22 ± 0.08	7.58 ± 0.20	0.78 ± 0.02	0.0602 ± 0.008	0.19 ± 0.03
	293	15.82	15.59 ± 0.65	0.99 ± 0.04	0.51 ± 0.09	-1.48 ± 0.20	14.9 ± 0.87	0.95 ± 0.05	0.0472 ± 0.002	-0.44 ± 0.05
	298	11.04	43.8 ± 2.2	3.97 ± 0.20	1.82 ± 0.26	-6.73 ± 0.47	37.4 ± 3.9	3.38 ± 0.35	0.0407 ± 0.002	-1.16 ± 0.21
4AP	280	21.86	0.65 ± 0.01	0.03 ± 0.00	0.06 ± 0.00	0.47 ± 0.01	0.79 ± 0.04	0.04 ± 0.00	0.154 ± 0.01	0.04 ± 0.05
	283	20.83	1.22 ± 0.02	0.06 ± 0.00	0.07 ± 0.01	0.43 ± 0.02	1.40 ± 0.07	0.07 ± 0.00	0.146 ± 0.01	0.19 ± 0.03
	288	22.48	3.54 ± 0.11	0.16 ± 0.00	0.10 ± 0.01	0.05 ± 0.04	3.64 ± 0.12	0.16 ± 0.01	0.128 ± 0.01	-0.05 ± 0.01
	293	23.85	10.54 ± 0.36	0.44 ± 0.02	0.37 ± 0.01	-1.99 ± 0.05	5.51 ± 0.56	0.23 ± 0.01	0.111 ± 0.006	0.16 ± 0.03
	298	24.33	7.04 ± 0.64	0.29 ± 0.03	0.05 ± 0.01	0.58 ± 0.13	7.28 ± 0.82	0.30 ± 0.03	0.098 ± 0.006	0.05 ± 0.05
NAM	280	23.48	1.33 ± 0.04	0.06 ± 0.0	0.15 ± 0.01	0.53 ± 0.03	1.49 ± 0.07	0.06 ± 0.00	0.25 ± 0.02	0.39 ± 0.03
	283	24.25	1.95 ± 0.05	0.08 ± 0.00	0.14 ± 0.01	0.50 ± 0.05	2.13 ± 0.08	0.09 ± 0.00	0.23 ± 0.03	0.23 ± 0.03
	288	24.92	3.13 ± 0.11	0.13 ± 0.00	0.14 ± 0.01	0.19 ± 0.05	3.46 ± 0.17	0.14 ± 0.01	0.208 ± 0.02	-0.08 ± 0.03
	293	26.37	5.95 ± 0.36	0.23 ± 0.01	0.10 ± 0.02	0.46 ± 0.16	6.25 ± 0.44	0.24 ± 0.02	0.185± 0.01	-0.04 ± 0.05
	298	27.22	7.53 ± 0.71	0.28 ± 0.03	0.09 ± 0.03	0.36 ± 0.27	7.95 ± 0.75	0.29 ± 0.03	0.160 ± 0.03	-0.17 ± 0.04

$C_SS_2 \leftrightarrow S_2$ model fitting for SEXSY experimental data

We fitted SEXSY experimental data using $C_SS_2 \leftrightarrow S_2$ model. Two studies were made to perform the best fitting: first, we fit normally with variable relaxation constant rates, and second by using fixed relaxation constant rates based on T_1 measurement of the free substrate at different temperatures. $R_f = 1/T_1$ (**Table S2**) is the relaxation constant rate for the free substrate in this model and R_e is the relaxation rate of the bound substrate in this model.

Table S4. Fitting parameters for all substrates using model $C_SS_2 \leftrightarrow S_2$ for SEXSY experimental data.

Substrate	T (K)	$\frac{[S]}{[C_SS_2]}$	*Model* $C_SS_2 \leftrightarrow S_2$, (with variable R_f values)				*Model* $C_SS_2 \leftrightarrow S_2$, (with fixed R_f)			
			k_d (s^{-1})	$k_{a'}$ (s^{-1})	R_f (s^{-1})	R_e (s^{-1})	k_d (s^{-1})	$k_{a'}$ (s^{-1})	R_f (s^{-1})	R_e (s^{-1})
Py	280	7.46	1.64 ± 0.06	0.22 ± 0.01	0.046 ± 0.02	0.63 ± 0.03	1.73 ± 0.06	0.23 ± 0.01	0.0926 ± 0.006	0.57 ± 0.02
	283	8.61	2.89 ± 0.16	0.34 ± 0.02	0.004 ± 0.054	0.80 ± 0.09	3.04 ± 0.13	0.35 ± 0.02	0.0855 ± 0.007	0.69 ± 0.05
	288	9.72	6.17 ± 0.31	0.63 ± 0.03	-0.048 ± 0.053	0.73 ± 0.14	6.40 ± 0.31	0.66 ± 0.03	0.0602 ± 0.008	0.49 ± 0.06
	293	15.82	10.75 ± 0.83	0.68 ± 0.05	0.19 ± 0.13	0.28 ± 0.35	10.45 ± 0.78	0.66 ± 0.05	0.0472 ± 0.002	0.65 ± 0.15
	298	11.04	32.7 ± 2.7	2.96 ± 0.25	0.71 ± 0.24	-2.85 ± 0.78	30.3 ± 3.0	2.74 ± 0.27	0.0407 ± 0.002	-0.41 ± 0.15
4AP	280	21.86	0.76 ± 0.02	0.03 ± 0.00	0.073 ± 0.006	0.43 ± 0.01	0.89 ± 0.04	0.04 ± 0.00	0.154 ± 0.01	0.33 ± 0.02
	283	20.83	1.64 ± 0.11	0.08 ± 0.01	0.095 ± 0.022	0.26 ± 0.06	1.74 ± 0.11	0.08 ± 0.01	0.146 ± 0.01	0.16 ± 0.03
	288	22.48	3.50 ± 0.10	0.16 ± 0.00	0.096 ± 0.008	0.07 ± 0.04	3.56 ± 0.12	0.16 ± 0.01	0.128 ± 0.01	-0.04 ± 0.02
	293	23.85	13.9 ± 2.7	0.58 ± 0.11	1.01 ± 0.31	-4.26 ± 0.93	8.2 ± 2.8	0.35 ± 0.12	0.111 ± 0.006	-1.22 ± 0.22
	298	24.33	41.5 ± 14.2	1.70 ± 0.58	1.99 ± 0.80	-10.9 ± 3.9	20.1 ± 10.5	0.82 ± 0.43	0.098 ± 0.006	-1.53 ± 0.32
NAM	280	23.48	1.11 ± 0.05	0.05 ± 0.00	0.130 ± 0.015	0.63 ± 0.03	1.31 ± 0.08	0.06 ± 0.00	0.25 ± 0.02	0.46 ± 0.04
	283	24.25	1.89 ± 0.07	0.08 ± 0.00	0.118 ± 0.014	0.66 ± 0.04	2.10 ± 0.13	0.09 ± 0.01	0.23 ± 0.03	0.41 ± 0.05
	288	24.92	6.04 ± 0.54	0.24 ± 0.02	0.288 ± 0.051	-0.92 ± 0.17	5.79 ± 0.55	0.23 ± 0.02	0.208 ± 0.02	-0.66 ± 0.07

	293	26.37	9.97 ± 0.84	0.38 ± 0.03	0.206 ± 0.04	-0.77 ± 0.21	9.89 ± 0.82	0.37 ± 0.03	0.185± 0.01	-0.66 ± 0.07
	298	27.22	48.7 ± 10.9	1.79 ± 0.40	2.37 ± 0.67	-13.5 ± 3.2	24.8 ± 9.8	0.91 ± 0.36	0.160 ± 0.03	-2.21 ± 0.35

EXSY protocols validation

EXSY protocols experimental comparison

Two protocols for EXSY data acquisition and processing were compared.

The first "one EXSY spectrum" protocol employed Eq. S37 for the calculation of exchange rate constants and relied on the acquisition of single EXSY spectrum with non-zero d_{mix}.

The second "two EXSY spectra" protocol additionally used the second EXSY spectrum acquired with $d_{\mathrm{mix}} = 0$ and employed EXSYCalc 1.0 software (MestreLab Research) for the data analysis.

The obtained results are presented in **Table S5**. It is clear that despite the weakness of the assumptions lying in the basis of single-spectrum protocol, the results are in good agreement with those of "two EXSY spectra" protocol. Therefore, it was concluded that "one EXSY spectrum" protocol can be safely used for the acquisition and processing of EXSY data for SABRE systems.

Table S5. EXSY data obtained using different protocols. The data were measured at 16.4 T NMR spectrometer.

[S] (mM)	[C_2S_2] (mM)	T (K)	EXSY protocol	d_{mix}, s	k_d (s^{-1})	$k_{a'}$ (s^{-1})
100	16.7	290	one EXSY spectrum	0.1	8.72	2.71
100	16.7	290	two EXSY spectra	0.1	8.61	2.74
100	16.7	290	one EXSY spectrum	0.4	7.76	2.24
100	16.7	290	two EXSY spectra	0.4	7.44	2.30
40	4.0	288	one EXSY spectrum	0.1	5.92	0.898
40	4.0	288	two EXSY spectra	0.1	5.83	0.905
40	4.0	288	one EXSY spectrum	0.2	5.30	0.768
40	4.0	288	two EXSY spectra	0.2	5.32	0.747

EXSY measurements at variable mixing time

The series of EXSY measurements were performed for the same pyridine sample at the same temperature of 298 K but with different mixing times d_{mix}. The results are presented in **Table S6**. The obtained exchange rate constants depend on the mixing time. Likely, for these conditions the correct rate constants were obtained at $d_{\mathrm{mix}} \geq 0.05$ s (the averaged value of 25.3 s^{-1} is in good agreement with 25.5 s^{-1} obtained using SEXSY).

Table S6. EXSY data was measured for Py at 298 K using variable mixing time d_{mix}. To account for the possible errors due to complicated integration of partially overlapping signals in EXSY spectrum, each spectrum was integrated twice, and rate constants were computed for each integration. In the table, the values averaged across two integrations are presented.

d_{mix} (s)	k_d (s^{-1})	k_a' (s^{-1})
0.02	17.4	1.85
0.03	19.8	2.09
0.04	21.7	2.34
0.05	25.1	2.44
0.06	25.6	2.40
0.07	25.1	2.33
0.10	25.2	2.31

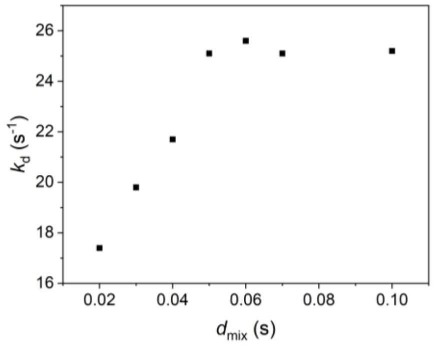

Optimal mixing time for EXSY

The SEXSY data was employed for the estimation of optimal mixing time for EXSY experiments using the formula[3]

$$d_{\mathrm{mix,opt}} \cong \frac{1}{R+\frac{k_{\mathrm{d}}}{2}+k'_{\mathrm{a}}} \quad \text{(Eq. S42)}$$

where R, k_{d} and k'_{a} were taken from the eigenvalue analysis of SEXSY data using $C_SS_2 \leftrightarrow S_2$ model. The calculated values are presented in **Table S7** along with the experimentally used values for comparison. The choice of mixing times in EXSY experiments was often suboptimal (although the estimated $d_{\mathrm{mix,opt}}$ values should be taken with care as Eq. S42 is an estimation).

Table S7. Estimated optimal mixing time values for the EXSY experiments ($d_{\mathrm{mix,opt}}$) and the values used in experiments ($d_{\mathrm{mix,exp}}$).

Substrate	T (K)	$d_{\mathrm{mix,opt}}$ (s)	$d_{\mathrm{mix,exp}}$ (s)
Py	280	0.598	0.500
Py	283	0.383	0.350
Py	288	0.225	0.160
Py	293	0.142	0.085
Py	298	0.066	0.050–0.100[a]
4AP	280	1.138	1.840
4AP	283	0.868	1.220
4AP	288	0.537	0.620
4AP	293	0.318	0.340
4AP	298	0.129	0.180
NAM	280	0.788	1.200
NAM	283	0.612	0.900
NAM	288	0.526	0.440
NAM	293	0.268	0.250
NAM	298	0.139	0.135

The range of mixing times was used for several EXSY measurements (see **Table S6**). The results measured at d_{mix} from 0.050 to 0.100 s were averaged and used for the analysis and presentation.

Enthalpies and entropies of activation

Eyring plots used for estimation of enthalpies and entropies of activation $\Delta H^{\ddagger}$ and $\Delta S^{\ddagger}$ (from ligand dissociation constants k_d presented in **Table 1**) are shown in **Figure S6**.

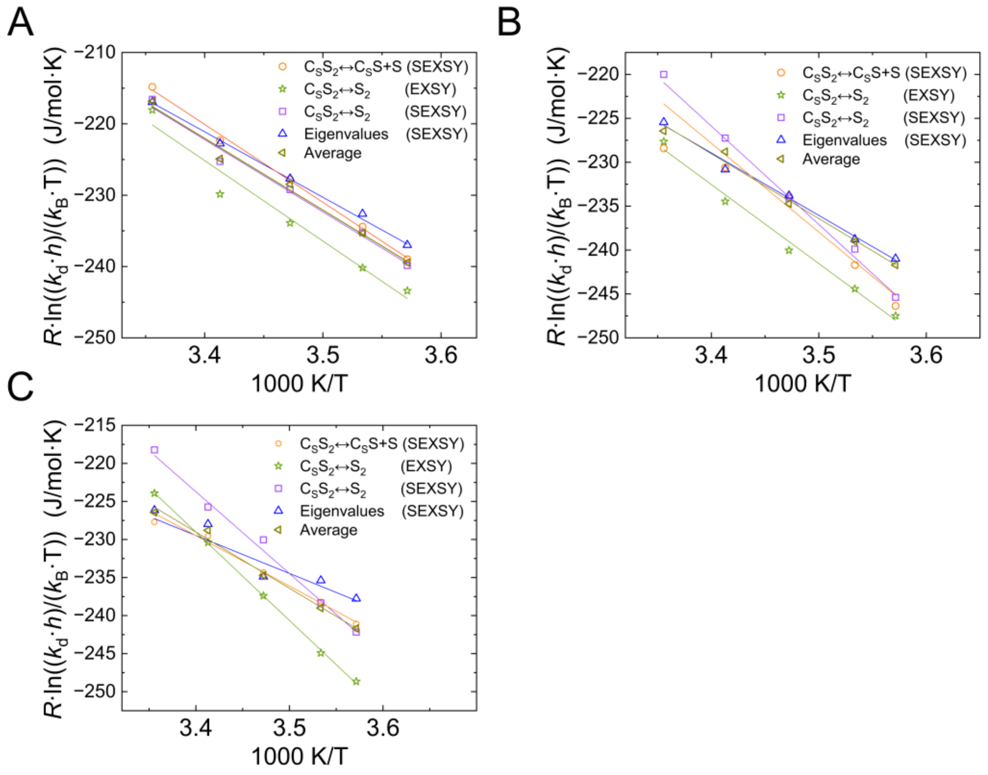

Figure S7. **Eyring plots for (A) pyridine, (B) 4-aminopyridine, and (C) nicotinamide**. Dissociation rate constants k_d were obtained with the models $C_SS_2 \leftrightarrow C_SS+S$ (SEXSY) (orange), $C_SS_2 \leftrightarrow C_SS+S_2$ (EXSY) (green), $C_SS_2 \leftrightarrow C_SS+S_2$ (SEXSY) (purple), eigenvalues analysis (blue), and average (left triangle). Here, these values are displayed in Eyring's coordinates.

Table S8. Enthalpies $\Delta H^{\ddagger}$ and entropies $\Delta S^{\ddagger}$ of activation for all substrates. $\Delta H^{\ddagger}$ and $\Delta S^{\ddagger}$ were obtained from dissociation exchange constants k_d (**Table 1**).

	From k_d averaged across all models		*Model* $C_SS_2 \leftrightarrow C_SS+S$		*Model* $C_SS_2 \leftrightarrow S_2$				Eigenvalues analysis: biexponential fitting (*Model* $C_SS_2 \leftrightarrow S_2$)	
			SEXSY		EXSY		SEXSY		SEXSY	
Substrate	$\Delta H^{\ddagger}$ (kJ/mol)	$\Delta S^{\ddagger}$ (J/(mol·K))	$\Delta H^{\ddagger}$ (kJ/mol)	$\Delta S^{\ddagger}$ (J/(mol·K))	$\Delta H^{\ddagger}$ (kJ/mol)	$\Delta S^{\ddagger}$ (J/(mol·K))	$\Delta H^{\ddagger}$ (kJ/mol)	$\Delta S^{\ddagger}$ (J/(mol·K))	$\Delta H^{\ddagger}$ (kJ/mol)	$\Delta S^{\ddagger}$ (J/(mol·K))
Py	101 ± 6	120 ± 22	109 ± 3	150 ± 11	112 ± 13	158 ± 45	101 ± 6	122 ± 21	91 ± 2	90 ± 7
4AP	21 ± 1	17 ± 19	101 ± 16	115 ± 56	90 ± 5	73 ± 18	112 ± 5	155 ± 18	71 ± 3	12 ± 11
NAM	74 ± 5	22 ± 16	66 ± 3	4 ± 13	116 ± 2	165 ± 5	107 ± 6	141 ± 21	49 ± 12	59 ± 43

Comparison with literature exchange rates

Table S9. Exchange rates k_{d} measured or estimated in literature for pyridine.

S9A: Technique: SLIC-SABRE, $\Delta H^{0‡}$ = 79.4 kJ/mol, $\Delta S^{0‡}$ = 52.9 J/(mol·K).[4]

T (K)	k_{d} (s^{-1})
273.15	2.1
278.15	4.1
283.15	7.6
288.15	13.9
293.15	24.8
298.15	43.6
303.15	75.2
308.15	127.5

S9B: Technique: SABRE-INEPT, $\Delta H^{0‡}$ = 76.2 ± 3 kJ/mol, $\Delta S^{0‡}$ = 33.3 ± 4 J/(mol·K).[5] As we identified in the text, these k_{d} values must be calculated as in Eq. 16 to represent more correct values.

T (K)	k_{d} (s^{-1}) published	k_{d} (s^{-1}) calculated as in Eq. 16
273.15	0.88	1.59
286.65	3.9	7.15
291.15	6.9	12.62
298.15	16.4	30.05
301.15	20.75	38.07

S9C: Technique: EXSY, $\Delta H^{0‡}$ = 93 ± 3 kJ/mol, $\Delta S^{0‡}$ = 97 ± 9 J/(mol·K).[6]

T (K)	k_{d} (s^{-1})
268	0.11
275	0.32
280	0.78
285	1.19
290	2.73
295	5.93
300	11.79

S9D: Technique: SEXSY, $\Delta H^{0‡}$ = 91 ± 2 kJ/mol, $\Delta S^{0‡}$ = 90 ± 7 J/(mol·K). (this work)

T (K)	k_{d} (s^{-1})
280	2.4 ± 0.1
283	4.2 ± 0.3
288	7.7 ± 0.4
293	14.1 ± 1
298	28.9 ± 1.6

References

(1) Ernst, R. R.; Bodenhausen, G.; Wokaun, A. *Principles of Nuclear Magnetic Resonance in One and Two Dimensions*, Repr.; The international series of monographs on chemistry; Clarendon Press: Oxford, 2004.

(2) Truong, M. L.; Theis, T.; Coffey, A. M.; Shchepin, R. V.; Waddell, K. W.; Shi, F.; Goodson, B. M.; Warren, W. S.; Chekmenev, E. Y. ^{15} N Hyperpolarization by Reversible Exchange Using SABRE-SHEATH. *J. Phys. Chem. C* **2015**, *119* (16), 8786–8797. https://doi.org/10.1021/acs.jpcc.5b01799.

(3) Perrin, C. L.; Dwyer, T. J. Application of Two-Dimensional NMR to Kinetics of Chemical Exchange. *Chem. Rev.* **1990**, *90* (6), 935–967. https://doi.org/10.1021/cr00104a002.

(4) Pravdivtsev, A. N.; Skovpin, I. V.; Svyatova, A. I.; Chukanov, N. V.; Kovtunova, L. M.; Bukhtiyarov, V. I.; Chekmenev, E. Y.; Kovtunov, K. V.; Koptyug, I. V.; Hövener, J.-B. Chemical Exchange Reaction Effect on Polarization Transfer Efficiency in SLIC-SABRE. *J. Phys. Chem. A* **2018**, *122* (46), 9107–9114. https://doi.org/10.1021/acs.jpca.8b07163.

(5) Pravdivtsev, A. N.; Yurkovskaya, A. V.; Zimmermann, H.; Vieth, H.-M.; Ivanov, K. L. Enhancing NMR of Insensitive Nuclei by Transfer of SABRE Spin Hyperpolarization. *Chem. Phys. Lett.* **2016**, *661*, 77–82. https://doi.org/10.1016/j.cplett.2016.08.037.

(6) Cowley, M. J.; Adams, R. W.; Atkinson, K. D.; Cockett, M. C. R.; Duckett, S. B.; Green, G. G. R.; Lohman, J. A. B.; Kerssebaum, R.; Kilgour, D.; Mewis, R. E. Iridium N-Heterocyclic Carbene Complexes as Efficient Catalysts for Magnetization Transfer from *Para* -Hydrogen. *J. Am. Chem. Soc.* **2011**, *133* (16), 6134–6137. https://doi.org/10.1021/ja200299u.
